# Tracking progress along the WHO Neglected Tropical Diseases Road Map to 2030: A guide to the Gap Assessment Tool (GAT) and results from the 2023–2024 assessment

**DOI:** 10.1371/journal.pntd.0013194

**Published:** 2025-07-01

**Authors:** Meritxell Donadeu, Theresa W. Gyorkos, Olaf Horstick, Patrick Lammie, Upendo J. Mwingira, Wyckliff P. Omondi, Joanna M. Pritchard, Jordan Tappero, YuYen L. Chan, Sophie Welsche, Junerlyn Farah V. Agua, Serge Madjou, Pamela S. Mbabazi, Supriya Warusavithana, Carlos A. Torres-Vitolas

**Affiliations:** 1 Faculty of Veterinary and Agricultural Sciences, University of Melbourne, Melbourne, Australia; 2 Department of Epidemiology, Biostatistics and Occupational Health, School of Population and Global Health, Faculty of Medicine and Health Sciences, McGill University, Montreal, Québec, Canada; 3 Heidelberg Institute of Global Health, Heidelberg University, Heidelberg, Germany; 4 NTD Support Center, Task Force for Global Health, Atlanta, Georgia, United States of America; 5 RTI International, Durham, North Carolina, United States of America; 6 Vector-Borne and Neglected Tropical Diseases Unit (VBNTDU), Ministry of HealthNairobi, Kenya; 7 Global Health, Bill and Melinda Gates Foundation, Seattle, Washington, United States of America; 8 Unlimit Health, London, United Kingdom; 9 Strategic Information and Analytics, Global Neglected Tropical Diseases Programme, World Health Organization, Geneva, Switzerland; 10 Eastern Mediterranean Regional Office, World Health Organization, Cairo, Egypt; Makerere University, UGANDA

## Abstract

The monitoring and evaluation (M&E) framework for the World Health Organization (WHO) Neglected Tropical Diseases (NTD) road map includes both quantitative and qualitative assessments of progress at key timepoints up to 2030. These assessments provide critical information to improve programmatic action and identify immediate research needs. Quantitative assessments process multi-sourced data on a predefined number of global indicators. Qualitative assessments are based on results obtained from implementing a Gap Assessment Tool (GAT). Informed by paradigm shifts detailed in the road map, a standardised methodology for the GAT has recently been developed. Methods include online public consultations and focus group discussions. GAT outcomes include a visualisation of progress in a ‘heat map’, a review of the current status of each NTD, and both disease-specific and cross-thematic recommendations for programmatic and research action. These outcomes provide valuable information not only for country NTD programmes, but also for global stakeholders, so that optimal efforts can be made to achieve established NTD road map goals, and ultimately, to reduce the burden of NTDs in at-risk populations. This manuscript provides a summary of the standardised GAT methodology and results from the implementation of the GAT in 2023–2024. Access is provided to the current status of each NTD with respect to four dimensions that were determined as priority in the 2019–2020 gap assessment (Diagnostics, Monitoring and Evaluation, Access & Logistics, and Advocacy & Funding). Results include a timely consensus on the critical and cross-cutting actions needed to improve respective programmatic impact towards eradication, elimination and control of NTDs globally and nationally.

## Author summary

We describe an innovative qualitative monitoring and evaluation assessment tool called the Gap Assessment Tool (the GAT) which was developed as a complement to routinely reported quantitative data, to periodically assess global progress towards goals set out in the World Health Organization’s Neglected Tropical Diseases’ Road Map to 2030. GAT methods include online public consultations, endemic country-based experiences and expert focus group discussions. Based on applying a standardised set of criteria, GAT outcomes include: a visualisation of progress in a ‘heat map’, a review of the current status of each disease, and both disease-specific and overarching recommendations for programmatic and research action. Selected results are presented to illustrate the value of these GAT outcomes, not only for country programmes, but also for global stakeholders, so that optimal efforts can be made to achieve road map goals, and ultimately, to reduce the burden of neglected tropical diseases in at-risk populations.

## Introduction

The World Health Organization (WHO) is committed to contribute to achieving the Sustainable Development Goals (SDGs) by 2030, for which neglected tropical diseases (NTDs) are the tracer for equity [1]. Among the many ways in which this commitment has been operationalised, is a global plan focusing on the eradication, elimination and control of NTDs (Box 1). This plan was endorsed by WHO member states as a road map in 2020, with interim benchmarks and end targets specified for each disease at specific timepoints to 2030 [2,3]. The road map reflected new thinking about how global communities and endemic countries, together with their diverse and dedicated stakeholders, could re-focus intervention efforts to more effectively and sustainably reduce NTD-related morbidity and mortality [4]. The impact resulting from a significant reduction in NTDs would strengthen contemporaneous efforts to attain the SDGs, while advancing related work for universal health coverage and primary health care. Three pillars of activity informed this re-focusing: 1. Accelerate programmatic action; 2. Intensify cross-cutting approaches; and 3. Change operating models and culture to facilitate country ownership.

Box 1 List of the NTDs (which considered 25 distinct disease groupings*), included in the Gap Assessment Tool (GAT) implemented in 2023-2024**

**Table:** 

Buruli ulcer	Human African trypanosomiasis (rhodesiense)	Scabies
Chagas disease	Leishmaniasis (cutaneous)	Schistosomiasis
Chikungunya	Leishmaniasis (visceral)	Soil-transmitted helminthiases
Chromoblastomycosis and other deep mycoses	Leprosy	Snakebite envenoming
Dengue	Lymphatic filariasis	Taeniasis and cysticercosis
Dracunculiasis	Mycetoma	Trachoma
Echinococcosis	Onchocerciasis	Yaws
Foodborne trematodiases	Other ectoparasites: tungiasis	
Human African trypanosomiasis (gambiense)	Rabies	

*Several NTDs combine distinct diseases for which separate assessments are required.

**Noma was officially recognized as an NTD in 2023 and has not been included in this GAT assessment.

To ensure accountability and sustainability of all three pillars along the timeline to 2030, a monitoring and evaluation framework was developed as a companion document to the NTD road map to define how progress should be measured [5]. Importantly, in addition to outlining the more traditional quantitative approach to monitoring and evaluation via the successful attainment of goals, targets and indicators, the framework also included a qualitative approach. By combining both types of approaches, recent evidence has unequivocally demonstrated that a more complete and comprehensive assessment can be obtained, and future activities subsequently improved [6,7].

An essential component in supporting the quantitative approach was the establishment of a Compendium of Indicators for NTDs [8], while an essential component in supporting the qualitative approach has been the development of a Gap Assessment Tool (GAT) which is described herein. The GAT was developed to gather timely experiential knowledge as well as cumulative research evidence in order to identify, and overcome, gaps which would hinder meeting road map goals. Importantly, the GAT adds substance to quantitative indicators by exploring reasons why an expected target or impact was, or was not, on track to be achieved, in order to best inform the programmes. The improved methodology in the newly developed GAT would provide greater standardisation, objectivity and replicability to monitoring and evaluation efforts. It would be a dynamic tool in assessing progress and identifying critical actions to fill identified gaps, thereby accelerating and streamlining programmatic activities. Its results would provide the research community with a timely appreciation of issues requiring urgent research attention. And, in reporting to the World Health Assembly, it would also provide a vehicle for inclusive and structured dialogue for engagement of multiple stakeholders and partners, increasingly supporting in-country ownership of programmes.

## Gap assessment 2019 prototype

The process of developing the GAT began in 2019, with wide-ranging discussions, facilitated by external consultants, to establish essential dimensions (or themes) to be assessed, for each NTD, and then for all NTDs, for a critical appreciation of each dimension across all NTDs [3]. A total of 11 dimensions were deemed essential (Box 2). Three dimensions were classified as having a *technical* component (comprising foundational knowledge requirements); five, as having a *strategy and service delivery* component (comprising essential aspects of NTD programmes); and three, as having an *enablers* component (informing action).

Box 2 **Cross-cutting dimensions assessed during the 2019 gap assessment for each NTD, and across all NTDs.**

**Table:** 

Component	Dimension
Technical	1. Scientific understanding 2. Diagnostics 3. Effective intervention
Strategy and Service Delivery	4. Operational and normative guidance 5. Planning, governance and programme implementation 6. Monitoring and evaluation 7. Access and logistics 8. Health care infrastructure and workforce
Enablers	9. Advocacy and funding 10. Collaboration and multisectoral action 11. Capacity and awareness-building

This 2019 gap assessment provided three outcomes: 1- A visual representation (‘heat map’) using a colour code (green, yellow, orange and red) to reflect perceived gaps for each of the dimensions, for each disease, obtained from a consensus among stakeholders from field level implementers and researchers. The stakeholders were identified by WHO, which also facilitated the consensus process; 2- the current status of each dimension for each disease; and 3- critical actions that needed to be addressed for each disease, and cross-cutting recommendations. These outcomes helped inform the building of the NTD road map [1]. Additionally, four of the eleven dimensions (i.e., Diagnostics, Monitoring and evaluation, Access and Logistics, and Advocacy and Funding) were specifically identified as being of high priority at the time, not only because of urgently needed attention but also because improved results would benefit multiple NTDs.

This initial gap assessment process was used as the base for the new standardised methodology developed during 2021–2023, which would then be replicated at specific timepoints up to 2030. A sub-group of the Monitoring, Evaluation and Research Working Group (MER WG) of the Strategic and Technical Advisory Group (STAG) of the Department of Control of NTDs at WHO was charged by WHO with improving the methodology to increase objectivity, standardisation and engagement of country programmes during future iterations of the assessment. The sub-group consisted of a secretariat, the GAT Steering Committee (some members of the MER WG), and other membership made up of WHO focal points, country representatives and invited experts, as required. Informed by the three paradigm shifts described in the road map, the new methodology was designed to provide clear and robust criteria to define the colour rankings for the heat map. It would establish a standardised process for information-gathering across diseases and dimensions to more objectively inform current disease status and, importantly, to propose critical actions to meet targets, ultimately reducing the burden of NTDs on vulnerable populations.

## Methods

### Developing a standardised process (2021–2023)

**Fig 1:**
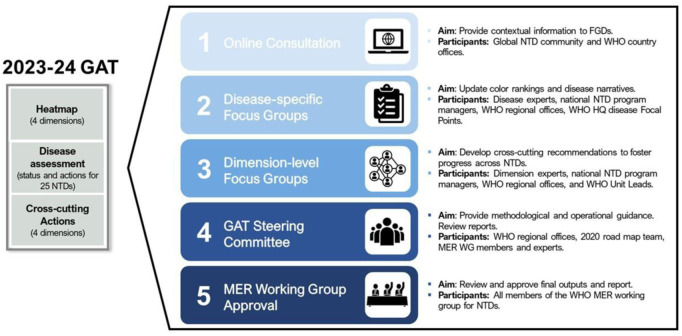
The cascade of GAT implementation steps.

The GAT sub-group’s first task was to provide the guidance for defining the standard criteria on which each of the eleven dimensions would be assessed -- for each NTD. A process task team, which included members of the MER WG and individuals with expertise in both qualitative research and NTDs who were recruited through the iCHORDS (Improving Community Health Outcomes through Research, Dialogue and Systems Strengthening) platform for social and behavioural researchers in NTDs, was formed to provide this guidance (Box 3). It also was charged with designing the cascade of steps to be undertaken to complete the entire process, from finalising the criteria, to implementing the GAT, and to reporting its results (Fig 1).

Box 3 **Guidance used to develop the standard criteria used to assess each dimension.**

**Table:** 

*Overall purpose* 1. Criteria should be written to assess progress and gaps at global level. 2. Criteria should be written in such a way that will both identify gaps AND track progress. 3. Wording should emphasize the use of tools/data/etc. at programme level, rather than presence/existence alone. (i.e., data are used for advocacy and to inform funding decisions instead of data are available) *Incorporating road map shifts* 4. Criteria should be drafted to embody the road map shifts (focus on impact, country ownership, and cross-cutting approaches) and other important road map principles (people-centered approaches, sustainability, equity) wherever possible. *Defining criteria for each colour ranking* 5. Criteria should be defined to represent the minimum requirements for each colour ranking, with red and green based largely on the high-level definitions included in the roadmap: ◦ Red = Critical action is required to reach road map target ◦ Green = There is no hindrance towards reaching road map target 6. For each colour ranking, all criteria pertaining to that colour must be met to achieve that colour. 7. Criteria need to be rigorous, but not so rigorous that progress will not be seen in the ranking when real progress is made, or that changes between colours take too long to achieve. 8. Criteria must be relevant to the baseline and keep in mind the road map targets. 9. The difference between colours should be as consistent as possible within dimensions to allow for steady progress through the colours. For example, avoid defining the criteria in such a way that progressing from red to yellow happens rapidly, but moving from yellow to green takes many years because the criteria for green are overly stringent. *Clarity and objectivity* 10. Criteria wording should be as precise and objective as possible. Words like “effective”, “clear”, and “adequate” should be defined through the criteria themselves (i.e., what are the criteria for an “effective” diagnostic) or written to include a brief description. *Standardization* 11. The goal should be to develop criteria that apply across all diseases to maximize standardisation.

The process of determining the cascade of steps was informed by reviewing the process used in 2019 and updating it to make it more robust and repeatable. It took into account a consultation process to engage NTD stakeholders and considered the best methods to use to apply the assessment criteria and recommend priority actions. This task team identified the following cascade of five implementation steps.

1. *Online Consultation:* Online surveys, one inviting participation from the global NTD community, and the other from country NTD programmes, to gather contextual information on all 11 dimensions, for each NTD.
2. *Disease-specific Focus Groups:* The purpose of this step is threefold: i) to conduct the colour assessment of each disease for the heat map, based on the improved criteria; ii) to review and update the ‘Current status’ and ‘Actions required’ for each disease; and iii) to provide input into the dimension-level focus group discussions for cross-cutting recommendations. Information from the two online surveys, as well as any relevant accessible quantitative data, is used to inform these focus group discussions. Each disease focus group discussion during the 2023/24 GAT lasted approximately 2 hours and followed the same process to agree on outcomes:
  - Colour rankings were assigned when disease-specific FGD participants agreed that minimum conditions for a given ranking were fulfilled.
  - Decision-making on rankings and narrative components relied on an iterative consensus building approach, by which initial views or positions were debated by participants until opinion exchanges no longer diverged.
  - Finally, a two-stage process was adopted to confirm outputs, with participants being asked to review draft reports after each discussion to add comments and confirm their agreement.
3. *Dimension-level Focus Groups:* The purpose of these focus group discussions is to review the information generated by the disease-specific focus groups with the intent of developing dimension-level cross-cutting recommendations. The consensus-building approach used for the disease-specific group discussions was also followed in these discussions during the 2023 GAT implementation, and the discussions also lasted approximately 2 hours each. Recommendations can inform both short-and-long term priorities for action with direct applicability to country NTD programmes. Results can also be used to more accurately pinpoint issues needing research attention.
4. *GAT Steering Committee:* This step ensures that there is consensus in reporting the final GAT assessment outcomes. The GAT steering committee also provides methodological and operational guidance when questions or challenges arise during the focus group discussions.
5. *MER WG Approval:* The final report of the GAT assessment is endorsed by this Working Group and is forwarded to the STAG of WHO for approval and disseminated, as appropriate.

Two virtual briefing meetings were held to orient all focus group participants on these five steps, during which detailed explanations and further clarifications of the GAT were provided where needed. This enabled participants to become well versed with the GAT thereby ensuring they were well prepared to participate in the focus group discussions.

## Finalising the standardised criteria for each dimension

Four task teams were established and charged with determining a parsimonious set of criteria for the four previously identified priority dimensions (i.e., Diagnostics; Monitoring and Evaluation; Access and Logistics; Advocacy and Funding). These task teams, of between four and eight members with pertinent expertise, were comprised of members from the MER WG and external experts (invited by WHO), as required. Proposed criteria were reviewed extensively by the GAT Steering Committee to achieve harmonisation and to ensure consistency before being finalised. This process was complex, and lengthy, as it required the assembly of groups of international experts who were required to meet (online) several times across many time zones.

To facilitate the establishment of the criteria for the remaining seven dimensions, a different, and more efficient, process was followed: the GAT Steering Committee itself developed the criteria, which were then peer-reviewed by the MER WG. This process incorporated lessons learned from the development of criteria for the initial four dimensions; applied a consistent approach to wording and distinction between criteria within each colour ranking for each dimension; tightened the timeline; and benefited from peer review provided, across dimensions, by the same critical mass of MER WG NTD experts. The criteria for the four priority dimensions are presented in Box 4, and the complete criteria for the eleven dimensions is available at S1 Table. Using these standardised GAT criteria, the status of each dimension for each NTD disease could be assessed. The overall colour ranking for a given dimension for a disease would be assigned depending on whether experts considered that the pre-established criteria for that dimension were, or were not, met. For example, for the diagnostics dimension, if disease experts considered that existing diagnostics for the disease under discussion (i) met the performance characteristics demanded by Target Product Profiles, (ii) supported at least one (but not all) current programme use cases, and (iii) met the quality standards established by WHO or other competent regulatory bodies, then the overall colour ranking assigned for the diagnostics dimension, for that disease, would be yellow.

Box 4 **Assessment criteria for dimensions: Diagnostics, Monitoring and Evaluation, Access and Logistics, Advocacy and Funding**

**Table:** 

Assessment Criteria	Colour ranking (Experts’ assessment)			
	green	yellow	orange	Red
1. Diagnostic tools meet the Target Product Profile (TPP) performance characteristics, or if TPPs not available, meet the ASSURED criteria	Yes	Yes	No	No
2. Diagnostic tools support programmatic actions for **[all/at least one/none]** current or future programme use cases	All	≥1	≥1	None
3. Diagnostic tools meet quality standards established by WHO or other competent regulatory bodies (FDA, WOAH, etc.)	Yes	Yes	No	No
**Dimension: Monitoring and Evaluation (M&E)**				
1.(…) endemic countries are implementing **monitoring and evaluation activities**, as per WHO disease-specific M&E framework	Most (>75%)	Majority (51–75%)	Some (25–50%)	None-few (< 25% or no framework)
2.(…) endemic countries report to WHO on **additional health indicators**, as per WHO disease-specific M&E framework	Most (>75%)	Majority (51–75%)	Some (25–50%)	None-few (< 25% or no framework)
3.(…) endemic countries that report to WHO on **road map indicators at the appropriate frequency for the disease**	Most (>75%)	Majority (51–75%)	Some (25–50%)	None-few (< 25% or no framework)
4.In (…) endemic countries, data collection processes for M&E activities have been **included within institutionalized health management information systems**, and data are adequately collected and reported to WHO	Most (>75%)	Majority (51–75%)	Some (25–50%)	None-few (< 25% or no framework)
5.If applicable for the disease’s public health goal, **disease-specific global guidance is available** on how to conduct **post-elimination surveillance** and sustain **validation or verification**	Yes	No	No	No
6.There is a **global system managed by WHO that collects, processes and stores data disaggregated** by age, sex, unit of assessment for road map and population health status indicators, as per WHO disease-specific M&E framework	Yes	No	No	No
7.Global guidance is available on **disease-specific indicators to integrate into countries’ Health Management Information System** (HMIS)	Yes	No	No	No
**Dimension: Access and Logistics**				
1.(…) endemic countries have an adequate **supply of products for preventive interventions to reach all relevant targeted populations at all levels**, when and where interventions are required	Most (>75%)	Majority (51–75%)	Some (25–50%)	None-few (< 25%)
2. (…) endemic countries have adequate **supply of products for individual patient care at all levels**, when and where required	Most (>75%)	Majority (51–75%)	Some (25–50%)	None-few (< 25%)
3. (…) endemic countries have **efficient supply chain** that allows the products to be available when and where required	Most (>75%)	Majority (51–75%)	Some (25–50%)	None-few (< 25%)
4. (…) endemic countries employ a fully functional and reliable **digital inventory management system** to ensure efficient management of products	Most (>75%)	Majority (51–75%)	Some (25–50%)	None-few (< 25%)
5. (…) endemic countries use **domestic funds for customs clearance or importation of donated or procured health products** in the past 12 months, where a waiver was not available	Most (>75%)	Majority (51–75%)	Some (25–50%)	None-few (< 25%)
**Dimension: Advocacy and Funding**				
1.(…) endemic countries have **national policies, strategies and plans in place** to ensure support for intervention activities	Most (>75%)	Majority (51–75%)	Some (25–50%)	None-few (< 25%)
2.(…) endemic countries have adequate **international funding** to make progress towards WHO 2030 road map targets	Most (>75%)	Majority (51–75%)	Some (25–50%)	None-few (< 25%)
3.(…) endemic countries have adequate **domestic funding** to make progress towards WHO 2030 road map targets	Most (>75%)	Majority (51–75%)	Some (25–50%)	None-few (< 25%)
4.(…) endemic countries have a **dedicated national budget line** for NTDs	Most (>75%)	Majority (51–75%)	Some (25–50%)	None-few (< 25%)
5.(…) endemic countries have **effective advocacy messaging to generate the required and sustained funding** (international and national)	Most (>75%)	Majority (51–75%)	Some (25–50%)	None-few (< 25%)
6.(…) endemic countries have a **sustainability plan** for the NTD programmes that incorporate strategies relevant for this disease	Most (>75%)	Majority (51–75%)	Some (25–50%)	None-few (< 25%)

NOTE: The assignment of an overall colour for a dimension, for each disease, is determined by appreciating the extent to which all criteria for that dimension have, or have not, been met.

In December 2022, the WHO NTD Department engaged a team of three methodologists, experts in both qualitative research and NTDs, to implement these steps in close collaboration with the WHO NTD Department and the MER WG GAT sub-group.

## Results of the 2023/2024 GAT implementation process

The new standardised GAT was implemented for the first time in 2023/2024. All implementation steps have been completed for all NTDs for the four initial priority dimensions first flagged in 2019. For the online consultation and the two focus group steps, the specific criteria shown in Box 4 were used for each of these four dimensions.

### Step 1. Online consultation

This step involved two online surveys, one public consultation aimed at the global health community and one at WHO country offices. Participants in the global survey were invited to provide their opinion on global-level trends concerning the four priority dimensions, while participants in the country survey reported on country-level activities of the dimensions. Information gathered on stakeholders’ perceptions of progress across diseases served as background information for subsequent focus group discussions.

Questionnaires for both surveys were produced by WHO in English and translated into the four other official UN languages (French, Spanish, Portuguese, and Arabic) to facilitate participation. A total of 205 individual respondents from different geographical areas participated in the global survey, while 67 country offices or programmes answered the country survey, mainly from the Global South.

### Step 2. Disease-specific focus groups

Discussions within each NTD disease-specific FGD used the newly standardised assessment criteria which had been finalised for each of the four priority dimensions. The discussions were informed by the information gathered via the online consultations. In addition, participants were provided with access to relevant WHO disease-specific background documentation, the NTD road map, the M&E Framework and the Compendium of Indicators [1,3,6].

**Fig 2:**
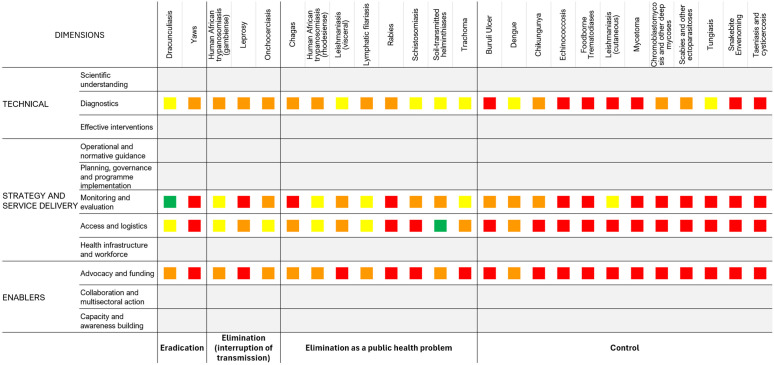
Updated 2023/2024 heat map for the initial four priority dimensions.

Twenty-two disease-specific focus group discussions were held between September 2023 and February 2024. Approximately 11 persons, composed of disease experts, programme managers from endemic regions, the WHO disease focal point, and WHO regional NTD focal points, participated in each focus group discussion. Summaries of the results by disease are available at: S2 Table. The summaries include the current status of each of the 20 NTDs (which include 25 actual disease groupings) for the four priority dimensions and the actions recommended to be implemented to improve programmatic impact. Based on consensus achieved in applying the standardised GAT criteria during the disease-specific focus group discussions, an updated heat map was produced (Fig 2).

### Step 3. Dimension-specific focus groups

The summaries of the four dimension-specific focus group discussions are available at: S3 Table. These discussions took place in May and June 2024. Approximately eight persons, composed of members of working groups identified within the WHO Department of the Control of NTDs, national programme managers, WHO leads, and WHO regional NTD focal points, participated in each dimension-specific focus group. The summaries provide extensive details regarding the inter-assessment changes in each dimension between 2019 and 2023/2024, and the respective positive and negative drivers of the respective changes. Salient conditions impacting current NTD programmes are listed and key recommendations are made for each disease and for cross-cutting actions. The following presents a brief overview of the main recommendations for the four priority dimensions which highlight opportunities for both programmatic action and research innovation to enable attainment of the road map goals by 2030.

#### Dimension: Diagnostics.

1. *Development of integrated diagnostics for multiple NTDs*: Access to diagnostics that can assess multiple NTDs can facilitate cross-cutting integration of programmatic activities while improving cost-effectiveness. This work needs to be aligned with programmes’ requirements to effectively support operations. This implies following the use-cases outlined in existing Target Product Profiles; adopting platforms that can be easily integrated into large-scale operations at primary care level; and updating operational guidance for programme managers that cover these new tools.
2. *Development of integrated diagnostics for zoonotic NTDs*: Diagnostics, for both humans and animals, could allow programmes to assess the presence of zoonotic disease reservoirs as well as the risk of reinfection in humans in a cost-effective manner. Monitoring of zoonotic diseases could be integrated into other NTD programmes. In addition to financial and technical support, the development of operational guidance is necessary to guide their use as part of integrated activities across multiple NTD programmes.
3. *Development of integrated diagnostics involving non-NTD diseases*: Established tests for non-NTDs may provide cost-effective and time-efficient opportunities for the development of integrated tests (e.g., skin diseases not listed as NTDs). Careful consideration should be given to the potential impacts of such developments on existing supply chains and manufacturing costs, to ensure their affordability and accessibility for all programmes.
4. *Strengthen advocacy efforts to support the development of integrated diagnostics:* Programmes should strive to coordinate their advocacy efforts to secure necessary resources. Mentioned strategies included emphasizing the health and economic benefits of integrated approaches and promoting more holistic advocacy narratives and frameworks (e.g., One Health). Early engagement with multiple potential manufacturers was considered useful to ensure long-term supply.
5. *Mainstream diagnostic activities within existing public health services:* NTD programmes can enhance their diagnostic capacity if they mainstream related operations within the national health system. This can provide greater access to laboratories and resources, as well as enhance programmes’ reach in peripheral areas. Country-level assessments are required to identify the most appropriate public services for integration and programmatic guidance on integration procedures is needed.
6. *Develop or strengthen regional (supra-national) laboratories*: The establishment of regional supranational laboratories for NTDs could help programmes to enhance their surge capacity, standardise provision across national and regional laboratories, expand access to facilities, and enhance quality assurance.
7. *Inter-sectoral collaboration for enhanced diagnostic capacity and development*: Collaboration with non-health sectors can enhance programmes’ diagnostic capacity through access to additional laboratories and resources; generate additional data for monitoring and evaluation, and planning (e.g., vector control); and grant access to resources to support the development of new or improved tests. Relevant sectors include Education, Water and Sanitation, Environment, Veterinary, and Agriculture. Engagement with the Education sector, in particular for higher education programmes and pre-service training, was considered valuable to help build in-country technical capacity across all healthcare levels.

#### Dimension: Monitoring and evaluation (M&E).

1. *Implement integrated M&E activities across NTD programmes with the broader health system and non-health sectors*: The implementation of joint M&E activities, such as mapping, impact assessments or surveillance, across NTDs programmes or in collaboration with the health system or other sectors (e.g., Veterinary and Agriculture) can widen programmes’ access to material and human resources and coverage among target populations, enhancing their efficiency.
2. *Harmonisation and integration of digital M&E systems and tools*: Timely access to up-to-date comprehensive M&E data is essential to assess programmatic progress at the country and regional levels, plan country-level programmatic activities, and inform international technical and policy debates. Experts supported the use, refinement, and expansion of existing NTD integrated data platforms; the development of new systems, when required; and the harmonisation of reporting formats across the sector as well as of M&E data systems for NTDs with those used by the public health system. Significant technical support to harmonise and integrate data systems and tools is required. Emerging digital tools, such as Artificial Intelligence, may be helpful in this regard.
3. *Data policies for integration and mainstreaming of M&E systems*: The integration of M&E operations could be further supported by ensuring that information from NTD programmes, countries, public health services and other sectors can be easily merged. Key data policies and measures recommended include: the harmonisation of M&E information requirements across NTD programmes, as well as with other sectors, and the establishment of data-sharing agreements with other sectors and between countries to monitor cross-border transmission.
4. *Operational research to support M&E integration*: The development of integrated approaches to M&E requires careful planning to maximize its potential benefits. Operational research initiatives can inform these developments, including assessing past and existing integration initiatives; gathering evidence of the tangible benefits accrued through such efforts; assessing the suitability of integrated approaches to address bottlenecks in M&E across NTDs; and assessing data compatibility between data systems.
5. *Developing or updating M&E guidance across NTDs*: Additional guidance is required to address emerging challenges affecting NTDs, as they progress towards road map targets. This includes linking initiatives producing post-elimination surveillance guidelines with those concerning the development of integrated diagnostics, to ensure their compatibility; developing standardised decision-making processes that mark when programmes move from control to elimination, and to eradication; developing guidance for the design and implementation of M&E activities in fragile, conflict-affected and vulnerable settings; and developing or updating M&E guidance for NTDs lacking suitable diagnostics.

#### Dimension: Access and logistics (A&L).

1. *Strengthen information systems to improve forecasting across programmes*: Good forecasting requires access to a variety of data sources. Recommended measures include improving access to health and population data, establishing feedback mechanisms across programmatic areas within NTD programmes (e.g., finances and M&E), developing a common platform for supply chain management and forecasting across NTD programmes, assessing the accuracy of existing forecasting tools to inform related improvements, and adapting validated forecasting tools from NTD and non-NTD programmes.
2. *Mainstream management and logistical operations into the national health system:* Integrating distribution and delivery operations within the public health system may increase programmes’ coverage and save costs through access to national distribution centers and local warehouses, for example. The integration of management information systems used by NTD programmes with those of the public health sector could also enable a more efficient administration of health products.
3. *Advocacy to support access and logistic systems*: Targeted financial support for A&L operations was considered necessary. Advocacy activities should highlight the role that management systems play in supporting programme activities and thus the need to strengthen such capacity to achieve the road map targets, address new threats, and support post-elimination activities. At the country level, advocating to governments to exempt programmes from paying taxes over the importation of drugs and other health products was considered key. Community engagement, in turn, was considered useful to generate demand-based estimates of treatment and intervention needs. Advocacy activities targeting NTD programmes were recommended to raise awareness about the benefits of integrating A&L operations across NTD programmes or with the public health system, and about the importance of including A&L experts in the development of guidance for other programmatic areas, since they can provide input on the practical and financial feasibility of recommended measures.
4. *Strengthen supply and procurement of NTD medicines and health products*: A&L operations require an adequate supply of quality health products that is sustained over time. Experts recommended that programmes coordinate with global stakeholders, including manufacturers, donors, and multilateral organizations, to expand the network of suppliers for key products as well as to establish standard requirements for medicines and health products procured outside donation schemes to ensure access to multiple reliable suppliers.
5. *Foster intersectoral coordination for zoonotic NTDs*: Experts considered it beneficial to seek integrating field operations for zoonotic NTDs with other relevant sectors, like Veterinary and Agriculture, to enhance programmes’ capacity to address the transmission risk posed by animals, access to resources, and the impact of interventions. They also recommended coordinating with country and global stakeholders (e.g., WOAH, FAO) to advocate for access to treatments and health products for both humans and animals.

#### Dimension: Advocacy and funding.

1. *Sector-level assessments for the development of guidance on effective advocacy*: Strategic alignment and coordination of advocacy efforts is necessary to enhance their effectiveness. Comprehensive assessments identifying funding gaps and priorities across programmes operating at the country, regional, and global levels, accompanied by mapping exercises of key stakeholders (funders and policy-makers), appear necessary. Such evidence can likewise inform the development of operational guidance on the design and implementation of advocacy efforts supporting integrated initiatives as well as on guiding advocacy planning for likely future scenarios.
2. *Strengthened programmatic structures to support advocacy*: To be effective, NTD advocacy campaigns require supporting organisational structures. Experts recommended mainstreaming and improving reporting mechanisms on funding and expenditure, to enhance transparency, enable cost-benefit analysis, and highlight funding gaps; providing additional support to region-level initiatives for inter-country coordination and information-sharing, and for greater WHO-HQ engagement; providing technical support to build local communications capacity and marketing expertise; and encouraging programmes to monitor epidemiological trends to seek opportunities for new partnerships. Including advocacy plans within national NTD master plans can be a useful mechanism to align advocacy activities along current key programmatic priorities and operations.
3. *Communication strategies to enhance advocacy efforts and facilitate integration*: Recommended approaches included using a ‘multiple disease elimination’ message to advocate for ‘last-mile’ activities; conducting evidence-based advocacy for elimination activities; generating work plans and cost-benefit estimates alongside advocacy campaigns to facilitate engagement with funders; showcasing NTD programmes’ contributions to other sectors (e.g., women’s health) to support inter-sectoral coordination; adopting synergistic messages and frameworks during high-level advocacy events; and promoting narratives focused on concrete specific subjects or operations (e.g., integrated treatment) for cross-cutting initiatives.
4. *Advocacy strategies for inter-sectoral collaboration*: Advocacy strategies to support inter-sectoral collaboration include: showcasing the contributions of NTD programmes to child and maternal health; mainstreaming programmatic activities within the public health system; positioning NTDs’ research and development agenda within existing initiatives to enhance pandemic preparedness and response across regions; showing how programmatic activities can serve as a platform for research and innovation; and promoting the inclusion of NTDs into debates on the impacts of climate change and urbanisation, to facilitate involvement in future related initiatives.

#### All four dimensions: overall observations.

The dimension-level focus group discussions showed certain commonalities in the conditions and steps required to support the implementation of cross-cutting recommendations.

- *Targeted advocacy for cross-cutting initiatives*: Advocacy and funding underpins the implementation of cross-cutting solutions -- which require close coordination across multiple stakeholders (NTD programmes, public health services, and non-health sectors). Advocacy is also required to mobilise funders and institutional backers, from national to global levels, to shift the funding landscape to be more receptive towards integrated initiatives.
- *WHO leadership for coordinated multi-level action*: Recommendations highlighted the importance of closer strategic alignment between initiatives at the country, regional, and global levels, chiefly through WHO engagement. High-level initiatives can foster international cooperation and governments’ political commitment -- which can then facilitate the implementation of cross-cutting initiatives at the local level.
- *Need for a more systemic approach to planning and implementation:* Cross-cutting solutions demand close coordination between programmatic areas from within NTD programmes themselves (e.g., Finance, M&E, Logistics) which are often perceived to work in a siloed manner. This would access to complementary information not typically consulted in the past as well as input from experts specifically consulted to address recognised challenges in programme implementation.
- *Programmatic guidance is required*: Initiatives integrating multiple NTD programmes, public health systems, or other sectors are still uncommon. To foster their adoption in the sector, programmatic guidance should be developed to inform programme managers about the strategies and procedures they should adopt for their design and implementation as well as the circumstances in which they are recommended.
- *Technical support is essential*: Cross-cutting recommendations highlighted the need for greater investments in building local technical capacity. There is a clear need for setting modern data systems in place, harmonising reporting systems, and adopting compatible indicators for NTD programmes as well as for the public health system and other sectors. Trained staff to process and generate quality information is essential.
- *Addressing sustainability is essential*: Sustainability remains an underlying concern across all dimensions. Cross-cutting recommendations aimed at widening access to resources and improving programmes’ efficiency should be examined against a backdrop characterised by limited access to long term funding and often constrained to certain components, like treatment. Significant concerns have been raised on how to maintain key operations over time as certain NTDs reach elimination.
- *Establish the role and contributions of NTD programmes for other sectors*: While there is agreement that intersectoral collaboration is required, efforts are still needed to develop a clear case for the inclusion of NTDs in intersectoral partnerships. Evidence can be gathered on the tangible benefits programmes can bring to other sectors, either in terms of impact, access to resources and stakeholders, or efficiency gains. Outlining clear roles and responsibilities in potential partnerships can support these efforts.

### Step 4. GAT steering committee

Beginning in December 2022, members of this committee met virtually, approximately every two weeks to provide oversight and guidance to the GAT implementation process.

### Step 5. MER WG approval

The final report of the 2023–2024 GAT assessment for the four priority dimensions was approved on August 27, 2024 and submitted to WHO on September 16, 2024.

## Discussion

### GAT strengths and limitations

The 2023/2024 implementation of the GAT provides useful experiential knowledge to assist endemic countries and WHO in monitoring and evaluating NTD programmes.

Foremost, it has provided a dimensionality to the more traditional quantitative approach to assessing progress. By identifying the *what, how* and *why* programmes are successful, or not, programmes can benefit in strategically adjusting policies, guidance and interventions in a more comprehensive, effective and timely manner. The new standardised methodology employed by the GAT process provides transparency as the criteria are clearly defined. Transparency makes the GAT process more robust as the well-defined criteria enable meaningful comparisons over time. The defined criteria also facilitate harmonization across diseases. The new methodology is inclusive, ensuring that multiple stakeholders, from global to local actors, can participate in a coordinated, constructive and consensus-building way in shaping programme implementation and sustainability. In this way, programme performance is enhanced and the likelihood of positive impact on the health of vulnerable populations is accelerated. Important non-negligible benefits accrue in terms of cost-efficiencies, both in terms of material and human resources; in terms of lessons learned, especially when applicable to other sectors and global concerns; and in terms of quality of life, by reducing NTD disease burden. Gaps identified, especially those where remedial action can benefit multiple NTDs simultaneously, can also be taken up by researchers, further enhancing opportunities for success. By implementing the GAT at critical timepoints along the road to 2030, targets and goals can become more achievable. Additionally, in the absence of in-person meetings due to COVID-related restrictions, the focus group discussions served an important role of maintaining connectivity and dialogue about NTDs among a broad variety of stakeholders who would have otherwise not met.

While the new standardised GAT process is more robust, the limitations to use it within a monitoring and evaluation framework are primarily in terms of time and cost. To ensure as wide a participation as possible, not only is support required for translation, but time is needed to organise and complete the online consultations and the focus groups. There is a cost to ensuring that the standardised GAT methodology is strictly implemented, analysed and interpreted. Independent expert facilitators in qualitative assessment are crucial to perform this task. Having a secretariat dedicated for this work is essential. Endemic countries in all WHO regions have contributed to the 2023–2024 assessment. However, due to limits in both time and financial resources, the experiences of many programmes could not be included. Consideration of these limitations is warranted to better prepare and implement the GAT for the remaining dimensions and for the next assessment timepoints.

## Conclusion

Implementation of the GAT in 2023/2024 has centered on the four priority dimensions identified in the initial gap assessment of 2019. Its results provide a current appreciation of NTD endemicity and programmatic efforts to address NTD morbidity and mortality. Critical actions are identified to improve respective control/elimination/eradication targets for each disease. Importantly, cross-cutting recommendations have also been identified which provide opportunities for positive impact which may previously have been overlooked or not considered. Completing the GAT assessments for the remaining seven dimensions will provide national NTD programmes with a more complete understanding of effective and efficient actions. This provides a solid baseline for comparisons of progress and success at key milestones along the road to 2030, to guide remedial strategic actions needed to be on course towards attaining set public health goals for the control, elimination and eradication of neglected tropical diseases.

## Supporting information

S1 Table WHO-GAT Assessment criteria for colour ranking assignation – 11 road map dimensions. Standardised assessment criteria used to provide colour rankings, by disease, for all eleven dimensions. These colour rankings are used to make the ‘heat map’.
(DOCX)

S2 Table WHO-GAT: Disease-specific summaries (Dimensions: Diagnostics, Monitoring & Evaluation, Access & Logistics, Advocacy & Funding). Summaries from the disease-specific focus group discussions are presented, for each disease, for four dimensions (Diagnostics, Monitoring & Evaluation, Access & Logistics, Advocacy & Funding).
(DOCX)

S3 Table WHO-GAT: Dimension-level (Cross-cutting) Recommendations. Recommendations from the dimension-level focus group discussions are presented, for each of the four dimensions.
(DOCX)
