# Supplementary material for: Tracking progress along the WHO Neglected Tropical Diseases Road Map to 2030: A guide to the Gap Assessment Tool (GAT) and results from the 2023–2024 assessment
Source: PLoS Negl Trop Dis. 2025 Jul 1;19(7):e0013194. doi: 10.1371/journal.pntd.0013194 (PMC12244624; doi:10.1371/journal.pntd.0013194)
Supplement: S1 Table — Standardised assessment criteria used to provide colour rankings, by disease, for all eleven dimensions. These colour rankings are used to make the ‘heat map’. (DOCX) [file pntd.0013194.s001.docx]

| WHO-GAT: ASSESSMENT CRITERIA FOR COLOUR RANKING ASSIGNATION11 ROAD MAP DIMENSIONS |
| --- |

| 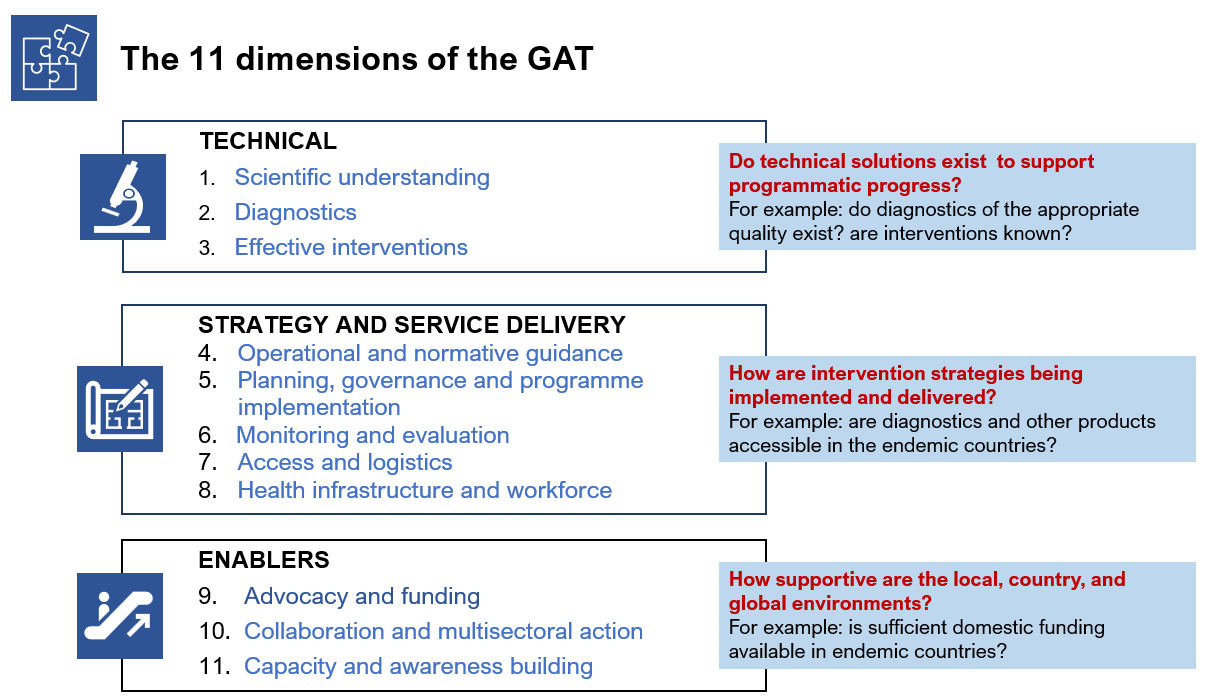 |
| --- |

The illustration above was generated using drawing tools and icons from Microsoft PowerPoint

| **Dimension 1: Scientific Understanding** | | | | |
| --- | --- | --- | --- | --- |
| **Road map definition:**   - Thorough understanding of disease epidemiology and pathology - No gaps in research that would hinder progress towards achieving targets - Understanding of the non-target effects of interventions (e.g. ancillary benefits, environmental effects) | | | | |
| **Assessment criteria** | **Colour ranking** | | | |
|  |  |  |  |  |
| 1. Understanding of disease epidemiology and pathology is sufficient to design effective interventions | Yes | Yes, but there are some gaps | No | No |
| 1. In (…) endemic countries, knowledge of geographic distribution of the disease (mapping) is completed according to the disease-specific mapping protocol and sufficient to guide the interventions required to reach road map targets. | Most | Majority | Some | None-few |
| 1. Non-target effects of interventions (e.g. ancillary benefits, environmental effects) are (…) | Known | Being assessed | Have not been assessed | Have not been assessed |
| **NOTES**:   1. Approximate range for categories:   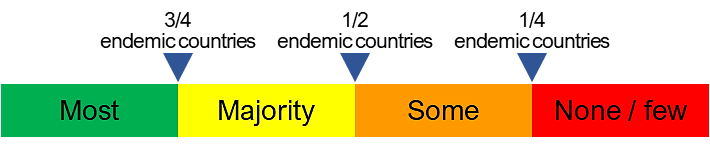 | | | | |

| **Dimension 2: Diagnostics** | | | | |
| --- | --- | --- | --- | --- |
| **Road map definition:**   - Availability of point-of-care diagnostics (where appropriate) usable at community level and in low-resource settings - Availability of effective, standardized, affordable diagnostics for timely detection, assessment of endpoints, surveillance | | | | |
| **Assessment criteria** | **Colour ranking** | | | |
|  |  |  |  |  |
| 1. Diagnostic tools meet the Target Product Profile (TPP)^a^ performance characteristics, or if TPPs not available, meet the ASSURED^b^ criteria. | Yes | Yes | No | No |
| 1. Diagnostic tools support programmatic actions for [**all / at least one / none**] current or future programme use cases. | All | At least one | At least one | None |
| 1. Diagnostic tools meet quality standards established by WHO or other competent regulatory authorities (FDA^c^, WOAH^d^, etc.) | Yes | Yes | No | No |
| **NOTES**:   1. Target Product Profiles (TPP): The TPPs are process tools that describe the essential features of a product (diagnostics in this instance), to guide researchers and manufacturers to develop solutions that meet the needs for specific use cases, so the final product is fit for purpose. 2. ASSURED criteria: Affordable, Sensitive, Specific, User-friendly, Robust and Rapid, Equipment-free, Deliverable. 3. FDA: US Food and Drug Administration. 4. WOAH: World Organization for Animal Health (formerly known as OIE). | | | | |

| **Dimension 3: Effective Interventions** | | | | |
| --- | --- | --- | --- | --- |
| **Road map definition:**   - Effective, affordable interventions for prevention, treatment, case management, rehabilitation and care. - Continued innovation and adaptation of interventions. | | | | |
| **Assessment criteria** | **Colour ranking** | | | |
|  |  |  |  |  |
| 1. Interventions for the prevention of disease are (…). | Known, effective, efficient and affordable | Known, but not effective, efficient or affordable | Known, but not effective, efficient or affordable | Not known |
| 1. Implementation of the preventive interventions is (…) due to the technical knowledge | Not limited | Not limited | Limited | Not known |
| 1. Interventions for treatment, case management, rehabilitation and care of patients are (…) | Known, effective, efficient and affordable | Known, but not effective, efficient or affordable | Known, but not effective, efficient or affordable | Not known |
| 1. Implementation of interventions for treatment, case management, rehabilitation and care of patients is (…) due to the technical knowledge | Not limited | Not limited | Limited | Not known |
| 1. Innovation and adaptation of the interventions is (…) | Regular | Periodic | Not present | Not present |
| **NOTES**:   1. Interventions refer to the **technical requirements** of drugs, combinations of drugs, vaccines, biologicals, and knowledge of how best to use them in the field. Diagnostics are not included here because they are covered under Dimension #2. The availability and implementation of interventions are also covered in other dimensions. | | | | |

| **Dimension 4: Operational and Normative Guidance** | | | | |
| --- | --- | --- | --- | --- |
| **Road map definition:**   - Clear definitions of endpoints and operational approach to achieve and sustain them. - Availability of technical guidelines, e.g. for validation or verification. - Equitable access to interventions (e.g. by disadvantaged, vulnerable and inaccessible populations). | | | | |
| **Assessment criteria** | **Colour ranking** | | | |
|  |  |  |  |  |
| 1. There is WHO normative and operational guidance to support (…) **preventive intervention** activities. | All | Some | Sufficient normative but insufficient operational | None |
| 1. There is WHO normative and operational guidance to support (…) **individual** treatment, case management, rehabilitation or care of patients. | All | Some | Sufficient normative, insufficient operational | None |
| 1. When applicable, there is (…) WHO normative and operational guidance to support validation, verification or certification activities. | Sufficient | Sufficient normative but insufficient operational | insufficient | Not |
| 1. WHO operational guidance is (…) updated to reflect innovation and adaptation of the interventions. | Frequently | Not | Not | Not |
| **NOTES**:   1. This dimension refers to GLOBAL guidance, while dimension #5 refers to COUNTRY guidance. 2. Normative guidance: Overall global level directive on attaining the Sustainable Development Goals using a road map for eradicating, eliminating and controlling NTDs (e.g.. the NTD Road Map 2021 - 2030 and its companion documents). 3. Operational guidance: Practical and detailed directives on implementing and evaluating intervention activities to achieve WHO 2030 road map targets. | | | | |

| **Dimension 5: Planning, Governance and Programme Implementation** | | | | |
| --- | --- | --- | --- | --- |
| **Road map definition:**   - Alignment and coordination of work among relevant stakeholders to achieve overall goals and milestones, based on a strategic plan. - Appropriate country governance and commitment for programme management and effective delivery. - Clear stakeholder responsibilities and effective, coordinated working processes to implement relevant interventions. - Effective planning and implementation at the country level. - Safe administration of treatment, and diligent monitoring and response to adverse events. | | | | |
| **Assessment criteria** | **Colour ranking** | | | |
|  |  |  |  |  |
| 1. (...) endemic countries have a functional national coordination body for NTDs that includes this disease | Most | Majority | Some | None-few |
| 1. (...) endemic countries are implementing control programs in all geographic areas in need of interventions. | Most | Majority | Some | None-few |
| 1. (...) endemic countries have national plans that include this disease. | Most | Majority | Some | None-few |
| 1. Where appropriate, (...) endemic countries have functional pharmacovigilance programmes that include this disease | Most | Majority | Some | None-few |
| 1. There is a global technical advisory group | Yes | Yes | No | No |
| 1. (...) endemic countries implement integrated NTD interventions | Most | Majority | Some | None-few |
| **NOTES**:   1. This dimension does not cover collaboration among stakeholders and involvement of communities, which is covered under Dimension #10. 2. A national coordination body may be a steering committee, working group, intersectoral planning group, national elimination committee or other body that provides national level oversight. 3. National NTD plans may also be called Strategic NTD plans. These plans should include all NTDs present in the country. In the criterion referring to pharmacovigilance, ‘where appropriate’ indicates that, for some programs, pharmacovigilance is not required because it does not include a drug intervention. 4. Integration between NTDs is captured here; collaboration with other health sectors and non-health sectors (education, WASH, etc) is covered under Dimension 10. 5. Approximate range for categories:   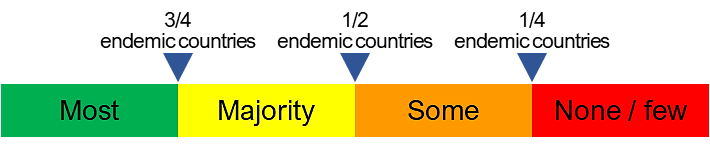 | | | | |
| **Dimension 6: Monitoring and Evaluation** | | | | |
| **Road map definition:**   - NTD monitoring and evaluation framework and mechanisms to monitor and report progress towards stated goals. - Standardized mapping and impact assessment for detailed view of disease epidemiology and progression. - Continuous, systematic, institutionalized collection, analysis and interpretation of health data disaggregated by age, gender, location, supported by strong data management systems and tools to assist in data interpretation for informed decision-making at all levels. - Strengthened and institutionalized surveillance for the disease, including post-validation and elimination surveillance. | | | | |
| **Assessment criteria** | **Colour ranking** | | | |
|  |  |  |  |  |
| 1. (...) endemic countries are implementing monitoring and evaluation activities, as per WHO disease-specific M&E framework. | Most | Majority | Some | None-few |
| 1. (...) endemic countries report to WHO on additional health status indicators, as per the WHO disease-specific M&E framework. | Most | Majority | Some | None-few |
| 1. (...) endemic countries report to WHO on road map indicators at the appropriate frequency for the disease | Most | Majority | Some | None-few |
| 1. (...) endemic countries, data collection processes for M&E activities have been included within institutionalized health management information systems, and data are adequately collected and reported to WHO. | Most | Majority | Some | None-few |
| 1. If applicable for the disease’s public health goal, disease-specific global guidance is available on how to conduct post-elimination surveillance and sustain validation or verification. | Yes | No | No | No |
| 1. There is a global system managed by WHO that collects, processes and stores data disaggregated by age, sex, and unit of assessment for road map and population health status indicators, as per WHO disease-specific M&E framework. | Yes | No | No | No |
| 1. Global guidance is available on disease-specific indicators to integrate into countries’ Health Management Information System (HMIS). | Yes | No | No | No |

| **NOTES**:   1. The framework provides guidance for country implementation of mapping and impact assessments, including survey methodology, guidance on what data programmatic decisions should be based upon, and indicator definitions (including level of disaggregation by age, sex, location etc.). 2. Health status indicators include morbidity and mortality and support the roadmap focus on measurement of impact. Additional information and examples of health status indicators can be found in the WHO Global Reference List of 100 Core Health Indicators. 3. Annually for diseases targeted for control and for elimination as a public health problem and for elimination of transmission; and monthly for diseases targeted for eradication 4. “Adequate” means satisfactory or acceptable in quality and quantity, to be further defined in the context of the expert discussions. 5. Approximate range for categories:   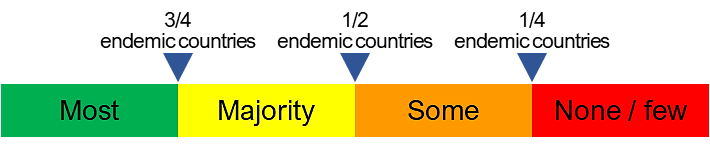 |
| --- |

| **Dimension 7: Access and Logistics** | | | | |
| --- | --- | --- | --- | --- |
| **Road map definition:**   - Adequate supply of affordable, quality-assured medicines, diagnostics and other medical products at all levels - Efficient supply chain for effective allocation and distribution of medicines, diagnostics and other medical products where they are needed while minimizing wastage and loss, e.g. with modern online inventory management systems | | | | |
| **Assessment criteria** | **Colour ranking** | | | |
|  |  |  |  |  |
| 1. (...) endemic countries have an adequate supply of products^a^ for **preventive interventions** to reach all relevant targeted populations at all levels, when and where interventions are required. | Most | Majority | Some | None-few |
| 1. (...) endemic countries have adequate^b^ supply of products for **individual** patient care^c^ at all levels, when and where required. | Most | Majority | Some | None-few |
| 1. (...) endemic countries have efficient supply chain that allows the products to be available when and where required. | Most | Majority | Some | None-few |
| 1. (...) endemic countries employ a fully functional and reliable^d^ digital inventory management system to ensure efficient management of products. | Most | Majority | Some | None-few |
| 1. (...) endemic countries used domestic funds for costs associated with customs clearance or importation of donated or procured health products in the past 12 months, where a waiver was not available. | Most | Majority | Some | None-few |
| **NOTES**:   1. Products refers to the affordable, quality-assured drugs, diagnostics, biologicals (e.g., anti-venoms, vaccines), consumables and other medical products required to implement effective population interventions or to provide adequate patient care. 2. “Adequate” means satisfactory or acceptable in quality and quantity. 3. Patient care includes treatment, case management, rehabilitation and any other care provided at the individual level. 4. A fully functional and reliable digital inventory management system means that health products at all levels are included in the national LMIS (logistics management information system) and used for current national and subnational planning purposes, including completion of product request forms (for example, Joint Request for Selected Preventive Chemotherapy Medicines or other WHO application tools). 5. Approximate range for categories:   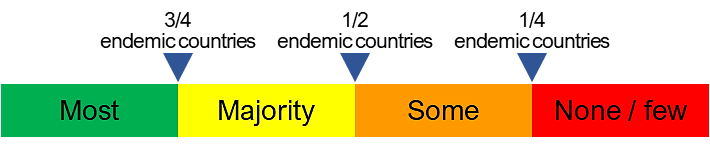 | | | | |
| **Dimension 8: Health Infrastructure and Workforce** | | | | |
| **Road map definition:**   - Robust health systems and primary health care infrastructure for delivering NTD interventions in models of integrated patient care - Laboratory capacity and network to support NTD programmes - Aptly skilled health care workers, including community volunteers and community healers, to meet clinical, entomological and community needs | | | | |
| **Assessment criteria** | **Colour ranking** | | | |
|  |  |  |  |  |
| 1. (…) endemic countries have a health care system with integrated patient care at (...) levels for early case management, MMDP, and referral for this disease, in (...) endemic areas. | Most  all  all | Majority  all  most | Some  some  some | None-few  some  few |
| 1. (…) endemic countries have laboratory capacity in endemic areas sufficient to support accurate and timely diagnosis for this disease | Most | Majority | Some | None-few |
| 1. (…) Endemic countries that have sufficient (...) health workforce at all levels of the health care delivery system, for this disease, including at the community level in (...) endemic areas. | Most  skilled  all | Majority  skilled  most | Some  but unskilled  some | None-few  -  most |
| **NOTES**:   1. Capacity building of healthcare personnel and staff is included in Dimension # 11. 2. MMDP: Morbidity management and disability prevention 3. Laboratory capacity refers to capacity to support interventions at the population or individual level 4. Approximate range for categories:   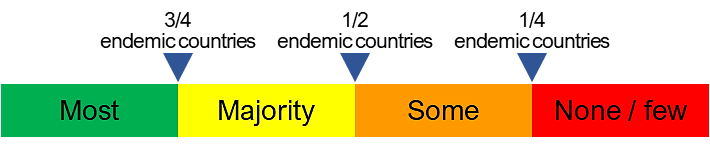 | | | | |

| **Dimension 9: Advocacy and Funding** | | | | |
| --- | --- | --- | --- | --- |
| **Road map definition:**   - Clear identification of funding gaps, and resource mobilization plans to address them. - Effective policy dialogue and advocacy to mobilize support for interventions in national and district health care delivery plans. - Adequate international and domestic funding to ensure sustainability of programmes, deployed with adequate lead time and consistency. | | | | |
| **Assessment criteria** | **Colour ranking** | | | |
|  |  |  |  |  |
| 1. (...) endemic countries have national policies, strategies and plans in place to ensure support for intervention activities. | Most | Majority | Some | None-few |
| 1. (...) endemic countries have adequate **international funding** to make progress towards WHO 2030 road map targets. | Most | Majority | Some | None-few |
| 1. (...) endemic countries have adequate **domestic funding** to make progress towards WHO 2030 road map targets. | Most | Majority | Some | None-few |
| 1. (...) endemic countries have a dedicated national budget line for NTDs | Most | Majority | Some | None-few |
| 1. (...) endemic countries have effective advocacy messaging to generate the required and sustained funding (international and national). | Most | Majority | Some | None-few |
| 1. (...) endemic countries have a sustainability plan for the NTD Programs that incorporate strategies relevant for this disease^a^. | Most | Majority | Some | None-few |
| **NOTES**:   1. Sustainability plans can be incorporated into the NTD Master Plan 2. Approximate range for categories:   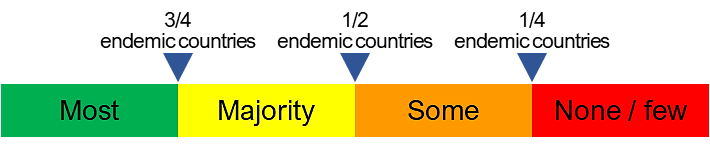 | | | | |

| **Dimension 10: Collaboration and Multisectoral Action** | | | | |
| --- | --- | --- | --- | --- |
| **Road map definition:**   - Collaboration among stakeholders across levels and sectors with clear accountability to ensure an effective, synergetic approach to delivering interventions. - Involvement of communities at risk and affected communities, e.g. in programme design. | | | | |
| **Assessment criteria** | **Colour ranking** | | | |
|  |  |  |  |  |
| 1. (...) endemic countries can document evidence of collaboration across all relevant sectors and/or partners that allow effective implementation of intervention activities. | Most | Majority | Some | None-few |
| 1. (...) endemic countries can document evidence of involvement of at-risk or affected communities in programme design. | Most | Majority | - | - |
| **NOTES**:   1. “Evidence” refers to official reports, work plans or documents that demonstrate intersectoral collaboration. 2. Relevant sectors include WASH, One Health, Integrated vector control, or other sectors that can contribute to positive outcomes. 3. Partners include non-governmental organizations, disease specific organizations, etc.. 4. Approximate range for categories:   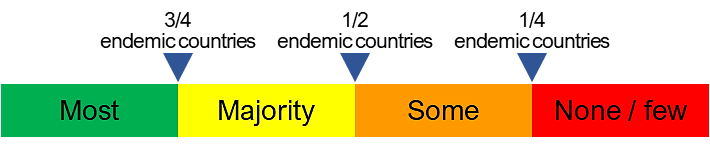 | | | | |

| **Dimension 11: Capacity and Awareness Building** | | | | |
| --- | --- | --- | --- | --- |
| **Road map definition:**   - Capacity-building to ensure high-performing programmes, e.g. pre-deployment and in-service training, transfer of skills from vertical NTD programmes to primary health systems, plans to handle health worker attrition and retirement, sharing uptake of best practices - Awareness-generation activities to educate and inform endemic communities, e.g. on behavioural changes, MDA scheduling, treatment and care options | | | | |
| **Assessment criteria** | **Colour ranking** | | | |
|  |  |  |  |  |
| 1. Most (>75%) endemic countries have adequate human resource training activities to scale up programme implementation for this disease to all endemic communities. | Yes, and is ongoing, specific, up-to-date and integrated into the country health’s system | Yes | - | - |
| 1. In endemic countries, communication materials promoting behavioural change required for programme success and sustainability for this disease are disseminated to endemic communities through all levels of the health care system. | Yes in most (>75%) endemic countries | Not necessarily | Not necessarily | Not necessarily |
| 1. Global guidance, including standardized materials for training health workers on all aspects of programme operations, is available for countries to adopt and adapt. | Yes | Yes | Yes | No |
| 1. (...) endemic countries conduct social mobilization activities required for programme implementation for this disease. | Most | Majority | Some | None-few |
| **NOTES**:   1. Social mobilization activities include behavioural changes, mass drug administration scheduling, treatment and care options 2. Approximate range for categories:   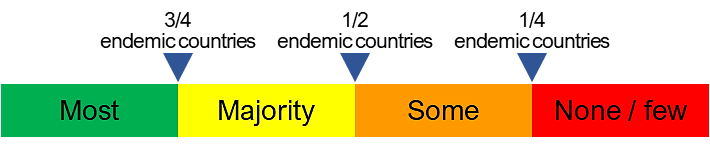 | | | | |
