# Supplementary material for: Tracking progress along the WHO Neglected Tropical Diseases Road Map to 2030: A guide to the Gap Assessment Tool (GAT) and results from the 2023–2024 assessment
Source: PLoS Negl Trop Dis. 2025 Jul 1;19(7):e0013194. doi: 10.1371/journal.pntd.0013194 (PMC12244624; doi:10.1371/journal.pntd.0013194)
Supplement: S2 Table — Summaries from the disease-specific focus group discussions are presented, for each disease, for four dimensions (Diagnostics, Monitoring & Evaluation, Access & Logistics, Advocacy & Funding). (DOCX) [file pntd.0013194.s002.docx]

| WHO-GAT: DISEASE-SPECIFIC SUMMARIES (Dimensions: Diagnostics, Monitoring and Evaluation, Access and Logistics, and Advocacy and Funding) |
| --- |

### ACRONYMS

AE Alveolar echinococcosis

CBM Chromoblastomycosis

CE Cystic echinococcosis

CL Cutaneous Leishmaniasis

CSL CSL Ltd (Australia)

DAT Direct Antiglobulin Test

DNA Deoxyribonucleic acid

DTU Discreet typing unit

ELISA Enzyme-linked immunosorbent assay

EPI Echo-planar imaging

FAO Food and Agriculture Organization

FGS Female genital schistosomiasis

FIND Foundation for Innovative New Diagnostics

F&E Face hygiene and environmental cleanliness

GAELF Global Alliance to Elimination Lymphatic Filariasis

GAVI Global Alliance for Vaccines and Immunization

GSK GlaxoSmithKline

HAT Human African trypanosomiasis

HIV Human Immunodeficiency Virus

HMIS Health management information systems

IDA Ivermectin plus Diethylcarbamazine and Albendazole

IRD International Relief and Development

ITM Institute of Travel Medicine

IVDR *In vitro* diagnostic medical devices regulation

IWGE Informal Working Group on Echinococcosis

LAMP Loop-mediated isothermal amplification

LF Lymphatic Filariasis

MDA Mass-drug administration

MDT Multi-drug therapy

MMDP Morbidity management and disability prevention

MDP Mectizan Donation Program

NCC Neurocysticercosis

NTD Neglected Tropical Disease

NYDA Dimeticones

PAHO Pan-American Health Organization

POC Point-of-care

PCR Polymerase chain reaction

PEP Post-exposure prophylaxis

PKDL Post Kala-Azar Dermal Leishmaniasis

RDT Rapid diagnostic test

RIG Rabies and Immunoglobulin Service

RNA Ribose nucleic acid

RPA Recombinase polymerase amplification

SAC School-aged children

SOP Standard Operational Procedure

ST Sporotrichosis

STH Soil-transmitted helminthiasis

TAS Transmission Assessment Survey

TB Tuberculosis

TC *Trypanosoma cruzi*

TPP Technical product profile

TGA Therapeutic Goods Administration

TT Trachomatous Trichiasis

TSOL *Taenia solium*

VL Visceral Leishmaniasis

UNEP United Nations Environment Programme

WASH Water Sanitation and Hygiene

WAHIS World Animal Health Information System

WIDP WHO Integrated Data Platform

WSP World Scabies Program

WOAH World Organization for Animal Health

WSP World Scabies Program

20WBCT 20-minute whole blood clotting test

### Buruli Ulcer (Target: Control)

| **Dimension** | **2023**  **ranking** | **Current status** | **Recommended actions** |
| --- | --- | --- | --- |
| **Diagnostics** | ◼ | - Diagnosis is done clinically or using laboratory techniques (direct microscopy, histopathology, culture, and PCR). - Early detection is essential in reducing morbidity. - *M. ulcerans* RDT, LAMP test, Biomeme (multiplex) PCR, and RPA test are being pilot tested in selected countries. - There is ongoing work in diagnostics. New methods are currently being developed and evaluated. | - Develop rapid diagnostic tools for use at public health center and community levels to enable early diagnosis, reduce morbidity and confirm cases. - Improve detection of viable *M. ulcerans* in wound samples to distinguish treatment failures and paradoxical reactions through methods such as mycolactone detection and 16S rRNA. |
| **Monitoring and Evaluation** | ◼ | - The majority of endemic countries currently report data according to the disease indicators. - Standard reporting forms including BU 01 and BU 02 are commonly used. - SOP request forms have been added to the standard reporting forms, and countries are moving towards an integrated Skin NTD register. | - Encourage reporting of data on Buruli ulcer in all endemic countries. - Enhance surveillance of Buruli ulcer in countries that are not reporting cases through integrated skin NTD reporting system. - Initiate micro-mapping of Buruli ulcer to identify overlaps with other NTDs and integrate approaches. - Monitor resistance to antibiotics phenotypically and through genetic markers. - Encourage focus on the development of adequate diagnostics to enable adequate performance of M&E activities. |
| **Access and Logistics** | ◼ | - WHO procures medicines and provides them to countries at no cost. - Governments and partners provide dressings and other supplies. - Support from some international partners has declined due to factors such as the COVID pandemic, while the chronic lack of prioritization in governments is leading to inadequate supply in some countries. - Access to preventative interventions remain a challenge at local levels remain. | - Ensure countries’ access to quality-assured medicines, which are currently provided by WHO with the support of donors. - Ensure adequate access to affordable improved dressings where required. |
| **Advocacy and Funding** | ◼ | - Political commitment through Yamoussoukro declaration (1998), Cotonou declaration (2009), and two Skin NTD meetings in 2023. - Donors and partners support implementation at country level. - The research community provides visibility and advocacy through mobilizing research resources. - Data to support updated and more efficient advocacy messages is lacking. | - Enhance political commitment among endemic countries and partners to mobilize funds and human resources. - Build community engagement and mobilization to support program implementation. - Sustain research funding for knowledge generation. - Support the creation of a global fund for NTDs as recommended during the Skin NTD meeting. |

### Chagas disease (Target: Elimination as a public health problem)

| **Dimension** | **2023**  **ranking** | **Current status** | **Recommended actions** |
| --- | --- | --- | --- |
| **Diagnostics** | ◼ | - There are various diagnostic tests, including serological, parasitological and molecular. Their use varies according to the phase of the disease, with parasitological and serological tests usually used for the acute phase and serological and molecular tests for the chronic phase. Tests used may also differ depending on the public health objective pursued. - A parasitological diagnosis by digital microscopy for hemoparasites has been developed but is still in the validation stage. - Marketed serological rapid diagnostic tests (RDTs) have been evaluated, but field validation remains to be completed. - Detecting markers of active infection is essential for clinical trials. - Performance of diagnostic tests may be impacted by t-cell clones/haplotypes. PCR may detect discrete taxonomic units (DTU) of TC, however there is no clear evidence of a relationship between TC DTUs and clinical manifestations or its congenital transmission, limiting their use as diagnostic or prognostic markers. - Current case diagnosis algorithms are based on two or three serological diagnostic tests. Discrepancies have been observed, however. Tests lack specificity/sensitivity. Diagnostic algorithms as well as antigens used for serological testing are currently being updated. - Ensuring patient adherence to conduct all tests required to confirm a diagnosis is difficult. - Some countries have incorporated new RDTs and automated tests such as those based on chemiluminescence. The incorporation of new diagnoses varies depending on the public health objective for which they are used. - There is unequal access to diagnostic tests between countries due to the costs associated with their acquisition and the varied state of the public health infrastructure in affected areas. | - Validate digital microscopy systems based on artificial intelligence developed to detect hemoparasites, including trypanosomes. - Complete the development of platforms for two RDTs, with high sensitivity and high specificity. - Validate the commercialized RDTs through multicenter field studies. - Develop tests to detect active infection and evaluate response to treatment. - Explore associations of clones/haplotypes of parasites that can be transferred congenitally and expand the antigen panel to detect multiple TC clones. - Adapt and verify with local samples the chemiluminescence tests developed for donor selection in blood banks and validate new algorithms using commercialized RDTs. - Complete the development and validation of new tests, such as LAMP molecular tests and multiplex PCR, to facilitate the development of automated Chagas diagnostics. - Strengthen quality control mechanisms for diagnostic tests. In addition to their approval by regulatory agencies or validation by laboratories, tests should be verified according to the specific requirements of each territory. Additional coordination with WHO can facilitate access to the necessary resources, e.g. immunoassays. |
| **Monitoring and Evaluation** | ◼ | - Methodological guides exist at the regional and national levels, with two areas currently being updated and completed by WHO: the frameworks for (i) estimating and reporting on indicators for epidemiological monitoring associated with control and elimination and (ii) assessing elimination and interruption of transmission, which is more advanced for vector control. - There is limited standardization of methodology for M&E indicators across countries: While currently 6 countries have adequate information and surveillance systems, others report to the WHO with data often combining estimates developed by different agencies and programs. The accuracy of such information remains uncertain. - Providing comprehensive up-to-date M&E data remains challenging due to the presence of various transmission mechanisms and public health objectives, which require multiple methodological approaches. Few countries have suitable information and surveillance systems to gather and process data for all interventions required to eliminate the disease. | - Complete the update of the methodological guides for the estimation and reporting of indicators for epidemiological monitoring associated with the control of Chagas and to monitor the interruption of transmission and elimination of the disease for all public health objectives. - To improve the quality of reporting, ensure that national programs apply the upcoming M&E protocols for evaluating control, elimination, and interruption of transmission. - Invest in the development and use of adequate information and surveillance systems among national programs, including the provision of technical support. - Target active screening to high-risk population groups. - Strengthen mandatory notification of acute and chronic cases. |
| **Access and Logistics** | ◼ | - There has been progress in the production and quality levels of medicines, with the addition of new production centers. Donations of nifurtimox, by Bayer, and benznidazole, by Chemo Ibérica, S.A., have been guaranteed until 2030. - There has been progress in universal health coverage of anti-parasitic treatment. However, difficulties remain at the community level, chiefly in rural areas. Recent migratory waves have generated new challenges to ensure access to treatment among groups that are not entitled to access public health services. - There are challenges in developing a planning system that ensures the availability of health products for preventive and therapeutic interventions at all levels and affected areas due to the lack of information available (e.g., population in need of treatment, stocks of medicines and other health products, trained personnel). | - Expand access to the health products necessary to detect and treat acute and chronic cases of Chagas, including congenital cases and complications associated with the disease. These resources must be available at the primary care level in the public health system. - Promote the development of adequate planning and administration systems to ensure access to the health products necessary to diagnose and treat acute and chronic cases of Chagas. Strengthening the development and use of information and digital management systems is necessary to achieve this objective. |
| **Advocacy and Funding** | ◼ | - Establishing International Chagas Day in 2020 has generated advocacy opportunities at the national and regional levels. - There have been advances in regional coordination for communication and resource mobilization initiatives across multilateral agencies and organizations (e.g., Chagas Coalition). - At the national level, advocacy efforts and achievements vary, depending on the institutional context (e.g., priorities of public and non-governmental entities). - Significant challenges remain to ensure domestic and international support to implement all recommended interventions. Support is often for short-term projects on specific topics. Accessing long-term comprehensive funding is difficult. - Programs require increased support due to recently increased oral transmission and more cases detected due to diagnostic advances. Available information systems are often unable to track and register required up-to-date data. | - Increase financing for the implementation of program activities. - Strengthen the advocacy work conducted by national programs to enhance their effectiveness, with technical support from multilateral organizations. |

### Chikungunya (Target: Control)

| **Dimension** | **2023**  **ranking** | **Current status** | **Recommended actions** |
| --- | --- | --- | --- |
| **Diagnostics** | ◼ | - Clinical challenge in differentiating chikungunya from dengue. - Serological tests including ELISA can confirm presence of antibodies. - RT-PCR methods are available to determine virus in blood but vary in sensitivity. - There is ongoing development of multiplex tests for patient management and surveillance. | - Develop more sensitive and specific diagnostic tests. Ensure they are affordable and accessible. - Systematically evaluate all tests to ensure their quality, especially POC tests. - Ensure the quality of testing in the field. - Encourage development of multiplex tests for confirmation of diagnosis and surveillance. - Develop rapid tests to distinguish chikungunya from zika and other arboviruses, among pregnant women. |
| **Monitoring and Evaluation** | ◼ | - Outbreaks have been reported in more than 80 countries across all regions. - State of surveillance and M&E systems vary by country. - Underreporting of M&E data is common. - Climate change and urbanization (poor design and management) have an impact on the disease. | - Develop surveillance and M&E systems in high-risk countries. - Develop digitized and early warning systems in all endemic countries. - Monitor resistance of vectors against insecticides. - Monitor presence and density of mosquitoes in areas of potential risk. |
| **Access and Logistics** | ◼ | - Regional stockpiles of spray equipment and insecticides in the Western Pacific Region. | - Consider developing regional stockpiles of spray equipment and insecticides in other endemic regions. - Ensure availability of high-quality molecular / multiplex tests. |
| **Advocacy and Funding** | ◼ | - Lack of data on the disease burden and economic costs of infection results in under-recognition of disease impact. - There is a greater need to finance new interventions, including vaccines. | - Increase commitment of resources from international donors, countries and non-governmental organizations for sustained interventions and patient care. - Develop and share resources among all stakeholders. - Create agreements with stakeholders that are developing preventive tools and strategies to ensure maintenance of original timelines. - Increase the funding for preventive interventions and early warning systems. - Ensure greater support for patient care, especially those with long-term disabilities. - Ensure greater intersectoral collaboration within countries and regionally. |

### Chromoblastomycosis and other deep mycoses (Target: Control)

| **Dimension** | **2023**  **ranking** | **Current status** | **Recommended Actions** |
| --- | --- | --- | --- |
| **Diagnostics** | ◼ | - Diagnosis based on clinical manifestation, epidemiological link, and demonstration of etiological agents from skin scrapings, biopsies or fungal cultures. - No rapid diagnostic test or any serological test available for chromoblastomycosis (CBM) and sporotrichosis (ST). - Early detection improves outcomes. - IMMY is developing a point of care test for sporotrichosis. | - Develop rapid diagnostic or serological tests to improve early detection at primary health care level. - Evaluate and standardize sporotrichin skin testing for diagnosis of ST. - Facilitate skin scraping and biopsy, and fungal culture and histopathology assessment of deep skin lesions. |
| **Monitoring and Evaluation** | ◼ | - No disease specific surveillance protocol or system, no standard indicators for M&E. - Guidance is being developed and the CURE-ID project is live. - Some states in Brazil have human sporotrichosis as a reportable disease, and national reporting for human cases is in development. | - Develop a surveillance guide with standard indicators and standardized case definitions. - Assess burden of chromoblastomycosis through active case detection and integrated surveillance of skin NTDs. - Establish M&E system, integrate with national health information system, and encourage the use of CURE-ID. - Describe true burden better, not just in terms of incidence and prevalence, but also in terms of disability and quality of life. |
| **Access and Logistics** | ◼ | - No donation of medicines. - Countries procure and manage their supply system; availability and affordability of antifungal medicines remain low. | - Ensure access to affordable and quality-assured itraconazole. - Provide affordable skin biopsy and sample processing for diagnosis. - Make drugs that are donated and indicated for HIV-associated fungal diseases also available for chromoblastomycosis. |
| **Advocacy and Funding** | ◼ | - Some organization and groups including the International League of Dermatological Societies (ILDS), Centers for Disease Control and Prevention (CDC), Drugs for Neglected Diseases initiative (DNDi), and the International Society for Human and Animal Mycology (ISHAM) are making advocacy, awareness-raising and capacity-building efforts. - Progress pertaining to advocacy and funding since 2019 has been limited. | - Ensure political commitment from endemic countries and donors/partners to mobilize funds and human resources. - Engage community and mobilize support for program implementation. - Mobilize funding for research on diagnostics, treatment, and M&E. - Push for formal inclusion within countries in terms of reportable disease and national medicines list. - Integrate with programs such as TB and other skin diseases. |

### Dengue (Target: Control)

| **Dimension** | **2023**  **ranking** | **Current status** | **Recommended actions** |
| --- | --- | --- | --- |
| **Diagnostics** | ◼ | - Dengue diagnostics are now included in WHO Essential Diagnostics List. - Rapid diagnostic tests exist for point-of-care diagnosis, but their quality is not assured. - There is ongoing work to develop multiplex tests for patient management and surveillance. Some are available but they are not affordable for programs. | - Improve quality assurance for point-of-care RDTs. - Develop molecular tests, including multiplex tests due to overlapping epidemiology of arboviruses, for confirmation of diagnosis, patient management and surveillance. - Improve affordability of existing and new diagnostic tools. |
| **Monitoring and Evaluation** | ◼ | - Underreporting of M&E data is common. - Reporting of M&E data often comes from sentinel sites only rather than from across the entire health system. - Insecticide resistance is emerging. - Climate change and urbanization (poor design and management) have an impact on the disease. - There is ongoing work to develop global guidance about the integration of disease-specific indicators into countries’ HMIS. | - Develop surveillance systems for preventive and rapid response interventions. - Ensure monitoring and reporting systems are established in all endemic countries. - Regularly monitor the presence and density of mosquitoes at sentinel sites using traps. - Monitor insecticide resistance. - Monitor the impacts of climate change and urbanization. |
| **Access and Logistics** | ◼ | - Regional stockpiles of spray equipment, RDTs (not quality assured), and insecticides in the WHO Western Pacific Region. - Improved access to materials for insecticide resistance monitoring. - New diagnostic doses for mosquitoes for WHO susceptibility bioassay determined and two new centers producing test papers opened in Brazil and India in addition to center in Malaysia. | - Initiate program for independent quality assurance of diagnostics. - Ensure availability of RDTs at all levels. - Encourage use of diagnostics in primary care settings for patient care and surveillance. Their use in pre-natal and maternal care settings is also recommended to distinguish Dengue from Zika. |
| **Advocacy and Funding** | ◼ | - Current levels of R&D funding have decreased. - There is a greater need to finance new interventions, including vaccines. | - Increase the commitment of resources from international donors, countries, and nongovernmental organizations. - Ensure greater intersectoral collaboration within countries and regionally. |

### Dracunculiasis (Target: Eradication)

| **Dimension** | **2023**  **ranking** | **Current status** | **Recommended actions** |
| --- | --- | --- | --- |
| **Diagnostics** | ◼ | - Laboratory microscopy and PCR are currently used. - Ongoing development of improved diagnostic tools for M&E, including PCR, serology, and microRNA. These tools are needed for post-eradication surveillance. - Ongoing development of the TPPs for environmental sampling and animal diagnostics. | - Complete development of serological field diagnostic test for humans and animals. - Complete development of field pond-side test for detecting *Dracunculus medinensis* DNA in copepods. - Complete development of further PCR and microRNA tests. |
| **Monitoring and Evaluation** | ◼ | - There is a need for improvement in program coverage among areas that are not known to be endemic but are at risk. - National programs monitor operational indicators, but better ways of measuring effectiveness of vector control intervention are needed. - Need for improved indicators for monitoring temephos application. - Theres is a need to incorporate indicators of animal infection in M&E reporting systems. Existing guidelines are currently being adapted. | - Ensure that monitoring systems are functional in non-endemic and formerly endemic areas that have not reported cases for a long time (e.g., particularly sensitive are the Central African Republic, Cameroon, and other at-risk countries bordering endemic countries). - Improve indicators for monitoring application of temephos. - Regularly monitor the quality of temephos application and resistance of Cyclops to temephos - Incorporate the veterinary system into indicators for reporting. - Ensure local capacity to identify and report cases of animal infection. |
| **Access and Logistics** | ◼ | - Adequate supply of temephos in endemic areas must be sustained. - Sufficient supply of ID cards (photos) and cash reward material must be maintained. | - Ensure that stock of temephos can be mobilized immediately to non-endemic and formerly endemic countries that reported cases or infection. - Ensure that country programs have the necessary human and material capacity to support the delivery of health products at all levels. |
| **Advocacy and Funding** | ◼ | - There are insufficient funds for program requirements. - There are delays in releasing funding at all levels. - Country programs regularly conduct advocacy activities. However, their success in securing resources vary. | - Ensure and sustain commitment for adequate funding from donors until eradication. - Increase advocacy in affected countries to sustain momentum, commitment, and funding nationally until eradication is achieved. - Eliminate delays in releasing funding at all levels. - Develop a return on investments for WASH in endemic areas. - Adopt a One Health approach to facilitate cross-sectoral collaboration and additional funding. |

### Echinococcosis (Target: Control)

| **Dimension** | **2023**  **ranking** | **Current status** | **Recommended actions** |
| --- | --- | --- | --- |
| **Diagnostics** | ◼ | - Imaging is currently the main diagnostic used in humans. - Serological tests are used for confirmation in humans but are not standardized. - Coproantigen-tests for canids are not adequately validated (CE and AE). - No practical screening test is available for livestock (CE). - Necropsy in dogs and livestock can be used as a diagnostic tool to evaluate control activities for CE. - Necropsy and SCT (Sedimentation and Counting Technique) of the intestines of red foxes can be a diagnostic tool to support surveillance activities of *E. multilocularis*. - Copro-PCR for diagnosis in definitive hosts and molecular and imaging tests for intermediate hosts (for both CE and AE) exist and are validated; however, they are mostly used for research and not currently practical in programmatic settings. | - Bring standardized, species-specific coproantigen and copro-PCR diagnostics for dogs (CE) and red foxes (AE) to market. - Develop Rapid Diagnostic Tests for diagnosis in humans and animals. - Define target product profiles and quality standards for diagnostics for both AE and CE. - Develop optimal diagnostics for humans and animals (for CE and AE). - Develop a subgroup within DTAG specific for zoonotic diseases including CE and AE. - Promote management of human CE patients based on WHO-IWGE Ultrasound Classification of CE. |
| **Monitoring and Evaluation** | ◼ | - Surveillance in humans and animals is weak in most countries. - Active surveillance in humans by population-based ultrasound screenings in limited resource settings. - There are ongoing efforts to define echinococcosis indicators (both AE and CE) and to include them in WHO toolkit for HMIS support. | - Assess baseline data at national and regional level to fully understand the scope of the challenge and evaluate control programs in resource-limited settings. - Set up active/passive surveillance systems for animals in highly endemic countries including meat inspections for CE. - Set up national disease registry systems for human cases and incorporate into surveillance. - WHO needs to prioritize and procure support for the development of an M&E framework and for data collection for severely neglected tropical diseases, including echinococcosis and to mandate the member states in endemic regions to send annual reports of disease surveillance. - Implement active surveillance in dogs by copro-PCR or copro-antigen ELISA tests (% data) and passive surveillance in livestock by meat inspection at slaughterhouse (% data). - Implement passive surveillance in humans by hospital records (incidence data). |
| **Access and Logistics** | ◼ | - Livestock vaccination for *E. granulosus* is now incorporated in the WOAH Terrestrial Code and Manual. Nevertheless, there is low demand due to low prioritization of livestock vaccination in the animal sector. - There is currently no donation of praziquantel and vaccines for treatment and prevention in animals. - Albendazole donation from GSK for human treatment recently secured, but requests from endemic countries have not yet been submitted. - Guidelines for patient care and management of CE taking into consideration the different structures of health systems are under development. | - Register EG95 vaccine for livestock in endemic countries (CE) and increase awareness of this intervention. - Set up a reliable supply chain for medicines and vaccines to ensure access. - Encourage endemic countries to invest in control, especially in treatment of dogs (AE and CE) and vaccination (CE). - Encourage endemic countries to integrate control measures with the control of other zoonotic diseases (e.g. leishmaniasis, brucellosis, rabies and bovine tuberculosis) on the same animal species (e.g. dogs, sheep, goat, cattle) in a One health approach, since this is expected to be more efficient and cost-effective (i.e. incorporate EG95 vaccine with other vaccines schedules in sheep). |
| **Advocacy and Funding** | ◼ | - Lack of political will and commitment for control efforts from governments in endemic countries. - Despite the development of the One Health approach, attention to endemic zoonotic diseases, including CE and AE is severely lacking in health systems. - Lack of commitment from international entities such as WHO/WOAH/FAO to include CE and AE in advocacy and funding schemes. | - Increase funding and support for animal and human health and strengthen the One Health approach. - Increase advocacy for implementation of active surveillance and control measures in animals and humans. - Encourage advocacy from WHO/FAO/WOAH/UNEP through a One Health lens for disease management and control activities. - Enhance support for patient care, early detection, and surveillance of human cases to improve the understanding of the disease burden. - Consider One Health as part of health system strengthening efforts. |

### Foodborne Trematodiases (Target: Control)

| **Dimension** | **2023**  **ranking** | **Current status** | **Recommended actions** |
| --- | --- | --- | --- |
| **Diagnostics** | ◼ | - Clinical diagnosis, parasitological techniques (e.g. detection of eggs in stool) or sputum tests are used. - Imaging is useful for morbidity assessment, but it is not available ubiquitously. - More sensitive serological techniques and molecular techniques (e.g., PCR) are used in research settings. - Several diagnostic tools are pending validation and are not yet available for use in the health sector (e.g., LAMP, dipstick and antigen tests). | - Evaluate and validate more field deployable and sensitive serological techniques, rapid tests and PCR. - Develop point-of-care diagnostic for intestinal, pulmonary, and liver flukes in humans. - Develop a rapid test to identify contamination of Fasciola in food sources. - Collaborate with industry to ensure validated tests being available and affordable in the health sector in endemic zones. - Streamline animal and human diagnostics, using a One Health approach. |
| **Monitoring and Evaluation** | ◼ | - Disease burden not well understood in humans and animals. - Assessments of the number of individuals at risk in each endemic country are not available. - Some countries are undertaking pilot surveys to assess the prevalence and burden of Foodborne Trematodiases. - *Paragonimus* screening is integrated with TB screening in some co-endemic areas. - New PAHO operational guidelines contain some information on M&E for fascioliasis. | - Estimate the number of individuals at risk by country. - Develop accurate surveillance, survey, and mapping methods, particularly layered with information on the environmental factors involved in infection. - Report changes in prevalence and incidence of liver cancer, cirrhosis and hepatic disease associated with control of these diseases. - Establish link between cancer, cirrhosis and hepatic disease register and hyper-endemic areas. - Link research institutions to the public health sector and local authorities. - Integrate mapping efforts with other NTDs, especially STH and SCH due to shared diagnostic (Kato-Katz). |
| **Access and Logistics** | ◼ | - Triclabendazole donation by Novartis continued and will include both *Fasciola* and *Paragonimus*. Discussions are ongoing for future commitments. - Countries can apply for donated triclabendazole tablets through WHO, however only few have done so. - Bayer has allowed praziquantel donations to be extended from taeniasis to foodborne Trematodiases, if there is excess supply. | - An efficient supply chain is established in most countries for other NTDs and could be used for foodborne trematodiases product supply. - Ensure product supply for animal treatment strategies. - Develop a list of pharmaceutical producers for the drugs recommended for animal fascioliasis (for all the parasite phases), including contact and prices. |
| **Advocacy and Funding** | ◼ | - No strong advocacy group is able to voice a global vision on these diseases. - Limited advocacy and funding due to lack of resources. - Limited support and engagement in terms of a One Health perspective from WOAH and FAO for Foodborne Trematodiases. - Data to underpin advocacy messaging is lacking. - PAHO has produced Operational Guidelines for elimination of human fascioliasis as a public health problem in the Americas. | - Create and sustain advocacy group for Foodborne Trematodiases. - Secure funding to tackle critical actions required to reach 2030 sub-targets. - Encourage advocacy from FAO/WOAH/UNEP through an inter-sectoral One Health lens for disease management, control activities and sustainability planning. - Enhance awareness of Foodborne Trematodiases among medical personnel and increase health education efforts in endemic zones. |

### Human African Trypanosomiasis – Gambiense (Target: Elimination (interruption of transmission))

| **Dimension** | **2023**  **ranking** | **Current status** | **Recommended actions** |
| --- | --- | --- | --- |
| **Diagnostics** | ◼ | - WHO have developed TPPs, which were recently published. - Screening is done with serological tests; parasite confirmation (in blood, lymph nodes or cerebrospinal fluid). - Screening tools are available but imperfect. Current confirmation tools are cumbersome. - Limited availability of tools to assess absence of the disease. - Different initiatives (DiTECT, FIND, IRD, ITM) are developing and evaluating new tools and protocols for screening and diagnosis. However, existing funding is insufficient. - Case-finding (active and passive) is the main activity for control and surveillance. | - Develop field-adapted diagnostic/detection tools (e.g., a simplified diagnostic that does not require confirmatory testing by microscopy). - Ensure independent, multicenter evaluation of new tools. |
| **Monitoring and Evaluation** | ◼ | - HAT Atlas is a helpful tool for planning and monitoring control and elimination activities. - Global indicators and methods for validation of HAT elimination as a public health problem are available. | - Use of data on disease distribution and case mapping tools to improve case-finding activities. - Improve the coverage of population screening activities to help identify at-risk populations (e.g. develop assessment methodology, transfer the process to country surveillance programs). - Secure financial and technical support for validation and verification. - Develop high-throughput tests to assess elimination and post-elimination surveillance on samples in a reference laboratory. - Reinforce surveillance through sentinel surveillance sites with trained staff and equipment. |
| **Access and Logistics** | ◼ | - Access to treatment is 100% ensured by donation from manufacturers and distribution is ensured by WHO until 2025. - Access to screening and diagnostics is not ensured and distribution of diagnostic tools is not systematic. - The future availability of diagnostic tools is uncertain | - Ensure availability and access of HAT diagnostic tools through involvement of manufacturers. |
| **Advocacy and Funding** | ◼ | - Important funding (Belgian Government, Sanofi, Bayer and the Gates Foundation) is guaranteed, but extension for long-term support is required. - Ownership of the elimination process and targets by endemic countries is weak. There is limited domestic funding available. | - Develop a long-term funding plan, including a campaign to mobilize resources to meet needs. - Maintain current support to ensure the sustainability of the current gains (e.g. lobbying to avoid donor fatigue). - Strengthen ownership of the elimination process and targets by endemic countries through advocacy to health authorities and heads of states in a context of decreasing numbers of cases. - Contribute to efforts advocating for inclusion of HAT-G as part of universal health coverage. |

### Human African Trypanosomiasis – Rhodesiense (Target: Elimination as a public health problem)

| **Dimension** | **2023**  **ranking** | **Current status** | **Recommended actions** |
| --- | --- | --- | --- |
| **Diagnostics** | ◼ | - No serological tests available and no ongoing research. - Diagnosis is done through parasite confirmation (in blood, lymph nodes or cerebrospinal fluid). - Stage of progression is determined by examining cerebrospinal fluid from lumbar puncture. - Expanded use of RDT for malaria has reduced the use of blood smear diagnostic technique. | - Develop new field-adapted tools to detect rHAT (e.g. RDT) to use in primary health care facilities (screening or diagnostic). - Maintain blood microscopy in clinical and laboratory algorithms, especially in areas of high risk for HAT-R, to counteract general decrease in use of microscopy for malaria suspicion due to the use of malaria RDTs. |
| **Monitoring and Evaluation** | ◼ | - Global indicators and methods for validation of HAT elimination as a public health problem are available. - Under-detection remains a concern. | - Targeting of case-finding activities to be improved by the use of data on disease distribution and case mapping tools. - Reinforce human case detection activities. - Secure technical support for validation process. - Reinforce surveillance through setting up sentinel surveillance sites with trained staff and equipment. |
| **Access and Logistics** | ◼ | - Access to treatment is 100% ensured through donated medicines that are distributed by WHO. | - If new diagnostics tools become available, their logistics and supply should be ensured. |
| **Advocacy and Funding** | ◼ | - Due to low prevalence, there is a significant funding gap for control, research, and monitoring & evaluation activities. - There is limited domestic funding available. - There has been an increase in the number of countries in contact with WHO to engage in advocacy and funding activities for HAT-R. | - Develop a long-term funding plan, including a campaign to mobilize resources to meet needs. - Maintain current support to ensure the sustainability of the current gains (e.g. lobbying to avoid donor fatigue). - Strengthen ownership of the elimination process and targets by endemic countries through advocacy to health authorities and heads of states in a context of decreasing numbers of cases. - Contribute to efforts advocating for universal health coverage. |

### Leishmaniasis – Cutaneous (Target: Control)

| **Dimension** | **2023**  **ranking** | **Current status** | **Recommended actions** |
| --- | --- | --- | --- |
| **Diagnostics** | ◼ | - Current diagnosis based on parasitological tests. - Clinical features lack adequate sensitivity, specificity and reliability in several endemic areas and laboratory diagnosis is not always available. - PCR is only available in reference laboratories and its use to support programs is limited. | - Develop affordable, more general, sensitive rapid diagnostic tests that can be used at health center and community levels and in all epidemiological situations. - Ensure that the process of registration and validation for new diagnostics is aligned with the technical requirements of endemic countries. |
| **Monitoring and Evaluation** | ◼ | - Most countries use aggregate data that does not allow for in-depth analysis or they struggle to report accurately. - Most countries lack comprehensive databases including disease and vector surveillance and control interventions data. | - Integrate electronic national databases with patient data for analysis, including data on vector surveillance and control interventions. - Ensure CL is made a notifiable disease and decouple roles dedicated to managing cases and reporting. - Strengthen the development of active surveillance systems and digital decision tools to allow for responsive interventions. - Collect data on mental health and quality of life impacts for all affected individuals. |
| **Access and Logistics** | ◼ | - Several high-burden countries face challenges in the procurement of necessary medicines, diagnostics, or physical treatment options for case management. - Several endemic countries lack the capacity to ensure the adequate distribution of health products in peripheral areas. | - Ensure availability of medicines and defined preventative and physical treatment for case management (procured or donated) in all countries through sufficient production and access. Undertake registration in endemic countries where this has not been addressed. - Improve access to and technical capacity for diagnosis and treatment in rural populations. |
| **Advocacy and Funding** | ◼ | - Key interventions, such as the provision of medical supplies or M&E, are fully reliant on external donors in high burden countries. - For countries with high burden of disease, there is insufficient domestic and international support. | - Increase domestic and international funding and improve other mechanisms to procure quality-assured medicines and diagnostics. - Develop national sustainability plans to ensure continued support in the future. - Train community and policy advocates to improve awareness, knowledge, and funding from all stakeholders. - Engage with a wider network of stakeholders, including additional multilateral organizations, to coordinate funding and advocacy activities at a global scale. |

### Leishmaniasis – Visceral (Target: Elimination as a public health problem)

| **Dimension** | **2023**  **ranking** | **Current status** | **Recommended actions** |
| --- | --- | --- | --- |
| **Diagnostics** | ◼ | - Sensitivity of diagnostic rapid tests may not be adequate for certain regions. - PCR is available in reference laboratories. - Besides microscopy, which needs invasive sampling (bone marrow, spleen), no deployable test of cure for VL and PKDL exists. - A second-line serological test (DAT) available in case rapid tests showed a negative result in a patient with suspected VL in East Africa. - Lack of VL patient post-treatment follow-up in certain countries. - The introduction of new IVDR regulations in EU are presenting a challenge to manufacturers in terms of compliance. - TPPs have been established and are due to be published soon. | - Need for individual rapid test to decentralize to the primary health care. - Develop more effective and user-friendly treatment and diagnostics, especially for East Africa. - Devise less invasive and highly specific tests to measure parasite level. - Develop less invasive test of cure for PKDL and VL. - Design and apply strategies and tools for patient tracking. - Further evaluation of the performance of other tests that are available but have not yet been validated is needed. |
| **Monitoring and Evaluation** | ◼ | - Lack of a standardized, integrated national and regional information system for all components, including patient follow-up, pharmacovigilance, and directing vector control. - Entomological surveillance needs additional attention in operationalization and implementation. - The first leishmaniasis vector control manual was published in 2023. - National guidelines for M&E have been published by PAHO for the Americas. | - Create a standardized integrated national, regional, and global information system for all components (disease surveillance, pharmacovigilance, vector and animal reservoir). - Conduct periodic independent external reviews; create incentives to improve implementation and monitoring. - Implement independent M&E of vector control activities to ensure quality and measure impact. |
| **Access and Logistics** | ◼ | - Poor forecasting of supplies and stock management (inventory) of insecticides, diagnostic kits and/or medicines. - Procurement policies may affect the quality of products purchased. - Some medicines are not available and/or affordable. - Medicines are available for selected countries through in-kind or cash donations from Gilead Sciences (AmBisome until 2025) and Sanofi (until 2025). - DNDI in collaboration with WHO discussed establishment of an excel-based forecasting template. | - Develop monthly reporting system to anticipate and avoid stockouts at health facility level. - Ensure accessibility of WHO quality-assured medical supplies through improved country procurement policies and capacity. - Improve access to affordable, quality-assured medicines - Develop innovative funding mechanisms, identify additional manufacturers, and secure pricing agreements. |
| **Advocacy and Funding** | ◼ | - Key interventions such as provision of medical supplies or M&E tools are fully dependent on external donors in several countries. - Even when countries have advocacy and funding for NTDs, support for visceral leishmaniasis is generally lacking. | - Increase sustained domestic funding to procure quality-assured medical health products for diagnosis and treatment. - Advocate for sustained funding and political commitment for research innovation. |

### Leprosy (Target: Elimination (interruption of transmission))

| **Dimension** | **2023**  **ranking** | **Current status** | **Recommended actions** |
| --- | --- | --- | --- |
| **Diagnostics** | ◼ | - Diagnosis of leprosy continues to be primarily clinical. - PCR is useful for diagnosis and surveillance of drug resistance but is only available in some countries (and usually in central laboratories). - Slit-skin smear is available for some cases (limited access). - Definition of relapses is still not agreed. Diagnosis is mainly done by exclusion. - Serology allows detection of infection but its utility to predict disease progression is limited. The tools are not available in most countries. | - Maintain and strengthen capacity for clinical diagnosis. - Enhance access to and capacity for slit-skin smear (across and within countries). - Wider use of slit-skin can enhance sample collection to support the further development of PCR and smear microscopy. - Develop a point-of-care test to confirm diagnosis and detect infection among those who were in contact with the disease. - POC tests are needed in areas where there is no health staff skilled in slit-skin diagnosis. - Develop and improve tools for diagnosis of sub-clinical cases. - Develop a standardized definition of leprosy relapses for the development of appropriate diagnosis. - Ensure that histopathological examination is available as an optional confirmatory diagnosis. |
| **Monitoring and Evaluation** | ◼ | - Integrated program reviews are occurring, with focus on reviewing progress in reaching the leprosy program targets. - Periodic monitoring for reactions is weak. - The referral system is very weak. - Post-MDT surveillance remains underdeveloped. - Roll-out digitalized case-based data management system is ongoing. - Mapping of cases is being introduced. | - Address training needs of health staff to ensure the expertise required to support M&E is available where required. - Improve notification systems to capture cases identified by private practitioners and community health workers. - Develop M&E guidelines specific to elimination of transmission. - Develop mechanisms to monitor adverse events. - Expand monitoring of antimicrobial resistance. |
| **Access and Logistics** | ◼ | - MDT medicines and clofazimine for reactions are available through Novartis donations, current commitment is until 2025. - Limited availability of second-line medicines. - Limited access to medicines to manage reactions. - Assistive devices to improve quality of life of persons affected by disabilities due to leprosy are mostly available but often with limited access and quality. - Limited access to social support and rehabilitative services, including counselling services for affected people. | - Bring drug supply chain systems in line with annual leprosy data. - Ensure supply of MDT, prophylactic medicines, second-line drugs and drugs to treat leprosy reactions. - Ensure availability of wound dressing materials. - Ensure access to assistive devices including customized footwear. - Ensure better access to leprosy services for women and girls. - Ensure adequate access to products supporting reconstructive surgery to respond to demand. - Ensure access of people with disabilities to rehabilitation services. - Enhance access to counselling services for affected people through primary health care services. |
| **Advocacy and Funding** | ◼ | - Many countries still depend on external sources of funding. - Where the prevalence of leprosy decreased, support for local programs often diminished. - High-level advocacy is essential to sustain interest in elimination of leprosy transmission but is currently insufficient. - Ongoing promotion of interest and investment in research: clinical, basic and operational research. | - Advocate with central and local governments to sustain and increase domestic funding despite ongoing decreases in prevalence and even in the post-elimination era. - Strengthen leprosy advocacy and funding activities to ensure it recovers its public health priority status after the COVID pandemic. - Mobilize domestic financing for country-level projects. - Continue periodic evaluation and high-level advocacy to inform ministries on progress and gaps and to increase engagement. - Advocate for policy based on evidence from research. - Make investment case for operational research in leprosy to support advocacy activities. - Ensure the human rights of leprosy-affected persons are respected. Existing discriminatory laws should be repealed. - Countries should take advantage of the WHO Global Leprosy Strategy 2021-2030 document for advocacy and funding purposes. |

### Lymphatic Filariasis (Target: Elimination as a public health problem)

| **Dimension** | **2023**  **ranking** | **Current status** | **Recommended actions** |
| --- | --- | --- | --- |
| **Diagnostics** | ◼ | - There are diagnostic tests available for recommended M&E. For *Brugia*, however, there is no reliable rapid test currently available. A *Brugia*-specific test is currently being evaluated. - *Loa loa* infection can create a false–positive result of the recommended LF antigen test. - WHO is in the process of employing an expert panel to review diagnostics for LF. | - Develop and improve diagnostic tests to inform decisions to stop-IDA, that do not cross-react with *L. loa*, are more field-reliable, and appropriate for surveillance. - Ensure reporting of issues with diagnostic tests for quality monitoring - Evaluate diagnostic tests currently available and in the pipeline to ensure capacity to meet the programmatic needs of the LF M&E Framework and future post-elimination surveillance guidance. |
| **Monitoring and Evaluation** | ◼ | - Lack of resources for M&E implementation in some countries. - There is limited surveillance among countries post validation. - Identification of focal residual infection can be challenging. - There has been progress to estimate endemicity in districts of previous uncertainty. - Lack of monitoring for the implementation of the essential care package for LF. - There is good procurement of diagnostics to support M&E activities for LF. | - Identify epidemiological settings where current thresholds for stopping MDA may not be sufficient; define new thresholds and develop survey methods. - Identify high risk areas, including urban settings, where MDAs might still be required. - Monitor the risk of reinfection through animals for *Brugia.* - Finish and disseminate guidelines for standards of surveillance and interventions to be sustained post-TAS and post-validation. - Integrate surveillance with NTDs, malaria or others where feasible. - Improve surveillance post-validation. - Develop and disseminate best practice guidance for management of acute attacks and lymphedema. |
| **Access and Logistics** | ◼ | - MSD, GSK and Eisai donate ivermectin, albendazole and diethylcarbamazine; global supply has been adequate to date. - IDA increased global demand of ivermectin. - MSD is meeting their commitment to Mectizan donations of up to 100 million treatments annually for IDA until 2025. - Efficiency of supply chain for LF elimination varies according to product (MDA supply chains usually being more efficient than those for MMDP). - Challenge to reach remote rural communities, islands, and conflict areas. - Continued low priority for MMDP in many countries is a concern. | - Improve planning, request sufficient medicines and diagnostic tests well in advance of program activities. - Make contingency plans for failed impact assessments or emergencies. - Make materials for lymphoedema management, hydrocele surgery and medicines to treat acute attacks available through the health system. |
| **Advocacy and Funding** | ◼ | - The Global Alliance to Eliminate LF supports advocacy/ resource mobilization with international and local donors. Additional activities might be needed to support elimination worldwide. - Limited prioritization and resourcing for MMDP and post-elimination surveillance in some countries - About US $18 million in funding is dedicated to R&D of LF between 2019-2021. | - Advocate the success and cost–effectiveness of interventions to facilitate governmental support. - Increase domestic funding and prioritization at all levels of government. - Advocate for the essential package of care for LF to be integrated into primary health care and included under UHC. - Ensure additional support to strengthen the GAELF. - Encourage sustained commitment for post-validation surveillance to prevent LF recrudescence. - Resources for improved diagnostics - Develop and disseminate best practice guidance for management of acute attack and lymphedema. |

### Mycetoma (Target: Elimination as a public health problem)

| **Dimension** | **2023**  **ranking** | **Current status** | **Recommended actions** |
| --- | --- | --- | --- |
| **Diagnostics** | ◼ | - The diagnosis is based largely on clinical presentation. - Causative organisms are identified through histopathology, culture of grains or through molecular identification directly on grains. - Ultrasound can be used for the identification of the disease and to differentiate actinomycetoma from eumycetoma. - Imaging can be used but requires trained technicians. | - Develop differential rapid diagnostic or serological tests to improve early detection at primary health care level. - Develop point of care test of cure. - Increase access to ultrasound to support diagnosis. |
| **Monitoring and Evaluation** | ◼ | - No disease specific surveillance protocol or system, no standard indicators for M&E, and no standardized case definition. - Guidance is being developed and the mycetoma case report form on the CURE-ID platform is live. - The only country reporting mycetoma data is Sudan. | - Establish standardized case definitions. - Assess burden of mycetoma through integrated surveillance of skin NTDs. - Integrate data collection with national health information systems and encourage the use of CURE-ID. - Describe true burden better, not just in terms of incidence and prevalence, but also in terms of disability and quality of life. |
| **Access and Logistics** | ◼ | - No medicine donations by manufacturers are currently in place. - Countries procure and manage their supply system. Availability and affordability of antifungal medicines remain low. - Fosravuconazole will be made available for early access in Sudan. | - Ensure access to medicines, including prequalification, procurement and affordability. - Ensure access to medicines and diagnostics (biological and ultrasound) in remote areas. - Make drugs that are donated and indicated for HIV-associated fungal and bacterial diseases also available for mycetoma. |
| **Advocacy and Funding** | ◼ | - Few partners and some mycetoma research institutions are exerting efforts to bring attention to the disease. - Some partners and governments have shown continued engagement in advocacy efforts. - Global Mycetoma Working Group, the Mycetoma Research Centre, Centers for Disease Control and Prevention, and DNDi are advocating and working towards improving knowledge through a clinical trial and a collaboration on a surveillance study. - The WHO Fungal Priority Pathogen List includes eumycetoma. - The lack of funding hampers research as well as the development of novel diagnostic tools. | - Ensure and sustain political commitment from endemic countries and donors/partners to mobilize funds and support human resources. - Increase partners’ commitment to improve access to medicines. - Increase country involvement in the global mycetoma working group. - Engage and mobilize communities to support mycetoma control programs. - Mobilize funding for research on diagnostics, treatment and M&E. - Push for formal inclusion within countries in terms of reportable disease and national medicines list. - Integrate with programs such as TB and other skin diseases. |

### Onchocerciasis (Target: Elimination (interruption of transmission))

| **Dimension** | **2023**  **ranking** | **Current status** | **Recommended actions** |
| --- | --- | --- | --- |
| **Diagnostics** | ◼ | - Serological and molecular tests are available but could be improved. - There are no standardized quality control and assurance systems for diagnostic tools used by programs in the field. - TPPs have been developed and published. - Ensuring supply of diagnostics in-country is challenging and often results in significant delays. | - Continue to improve performance of existing diagnostics (e.g., OV 16 ELISA) and evaluate diagnostics in development. - Devise a confirmatory diagnostic(s) for use in low-prevalence settings that could assist with mapping, MDA stopping decisions, and surveillance. - Develop a diagnostic strategy for the identification of *Loa loa* infection intensity. - Fast-track accessibility of new diagnostics to countries, e.g. qPCR. - Develop SOPs and standardized training materials for the use of diagnostics in the field. - Establish clear procurement systems and quality control mechanisms for new and existing diagnostics. |
| **Monitoring and Evaluation** | ◼ | - Mapping of hypoendemic areas in Africa is incomplete. - Lack of funding for M&E activities hampers countries’ ability to implement M&E activities according to existing guidelines and strategies. - Strategy for post-elimination surveillance and verification is being developed. - Onchocerciasis elimination mapping guidelines have been developed and are under review for publication. - Ongoing efforts for integrated M&E strategy for onchocerciasis and lymphatic filariasis (I-TAS). | - Complete the review of onchocerciasis elimination mapping strategy. - Develop and disseminate protocols for standardization of mapping to ensure consistency of data. - Improve mapping and sampling in *Loa loa* co-endemic areas to allow for granular treatment approaches. - Close data gaps in hypoendemic areas through development of more easy-to-use tools. - Ensure that M&E guidance reflects new tools and revised thresholds. |
| **Access and Logistics** | ◼ | - Strong supply for medications donated by Merck through the Mectizan Donation Program. - Supply of treatment is adequate for mass-drug administration (MDA) activities, but access is limited at the health facility level. | - Ensure countries have the appropriate plans and systems in place to access and distribute new medicines as they are being developed. - Develop a plan to facilitate the addition of new medications to the supply chain as they become available. - Strengthen digital systems to facilitate planning and monitoring of MDA activities, especially at the ‘last mile’. - Improve planning, procurement, and logistics systems to ensure countries have access to needed diagnostics available now and in the future. |
| **Advocacy and Funding** | ◼ | - Most programs are dependent on external donor support. - Country leadership of their programs and mobilization of investment remains challenging. - Efforts to institute sustainability plans are ongoing. - The establishment of GONE (Global Onchocerciasis Network for Elimination) is enabling additional support for program activities. | - Develop advocacy plan and continue advocacy to ensure donor support and increase domestic financing to ensure sustainability. - Ensure and maintain support for GONE to accelerate advocacy and funding. - Develop alternative advocacy and funding strategies to ensure a wide portfolio of interventions to accelerate progress towards elimination. |

### Rabies (Target: Elimination as a public health problem)

| **Dimension** | **2023**  **ranking** | **Current status** | **Recommended actions** |
| --- | --- | --- | --- |
| **Diagnostics** | ◼ | - Well-established diagnostic protocols are available. - Comparative assessments of various diagnostics are ongoing. | - Validate the postmortem diagnosis of rabies in animals (e.g. rapid diagnostic test) to improve post-bite treatment. - Prioritize developing rapid diagnostic tests for humans to detect more cases post-exposure. |
| **Monitoring and Evaluation** | ◼ | - WIDP module on rabies has been finalized. - WOAH World Animal Health Information System (WAHIS) for animal rabies reporting is in place and has been updated; however, it is not used for M&E. - Unclear who is responsible for rabies M&E and data collection at country level, a designated focal point is often lacking, and rabies is not well integrated into NTD reporting systems. - Global guidance and data systems are available. | - Improve roll-out of WHO guidance and data quality nationally and sub-nationally to ensure compliance with reporting. - Strengthen surveillance e.g. introduce indicator of suspicious death after bite, develop process for collecting samples. - Appropriate investment is needed to cover all M&E needs. - Push countries to complete their national strategic plans and to apply for WOAH dog control programs. - Encourage countries to develop feasible scale-up plans and funding after piloting M&E activities. - Support digitization of data collection and M&E systems at country level. |
| **Access and Logistics** | ◼ | - There is adequate global production capacity for rabies vaccines. - Demand forecasting and management of vaccines is weak, leading to stock-outs. - The WOAH rabies Vaccine Bank is operational, although capacity and supply are dependent on demand. - Donation of vaccines is pledged, however operational costs and technical support are not covered, which prevents country programs from effective roll-out. | - Ensure availability of quality-assured human and animal vaccines, e.g. collect data on vaccine/RIG use to forecast demand and inform the vaccine procurement system. - Integrate PEP into EPI systems to strengthen the supply chain. - Develop innovative approaches to improve delivery systems and ensure timely access to PEP and dog vaccinations. - Improve systems to identify patients with rabies to aid the prioritization of vaccine application. |
| **Advocacy and Funding** | ◼ | - Strong investment by GAVI in rabies vaccines for humans and animals. - United Against Rabies donor landscaping continues to increase the profile of rabies. - World Rabies Day remains a good initiative for rabies education. | - Secure investment for mass dog vaccinations to complement the GAVI initiative. - Strengthen country-level commitment and political will to rabies; ensure demand from communities for rabies services is recognized. - Push for the international community to respond to the demands of the countries rather than applying top-down funding. - Integrate into other NTD programs to effectively use available funding. - Showcase rabies as an initiative and proof of concept for operationalizing One Health approaches. |

### Scabies and other ectoparasitoses (Target: Control)

| **Dimension** | **2023**  **ranking** | **Current status** | **Recommended actions** |
| --- | --- | --- | --- |
| **Diagnostics** | ◼ | - Methods exist for screening and individual diagnosis but lack point-of-care confirmatory diagnostics. - New international consensus criteria 2020 will facilitate programmatic screening. - TPPs have been established and published. - Ongoing discussions around revising and simplifying the criteria for clinical algorithms to be used in the field. | - Validate clinical diagnostic algorithms for programmatic use in the field. - Develop population-level diagnostics for program activities, including epidemiological assessments, and facilitate integration with other NTD programs. |
| **Monitoring and Evaluation** | ◼ | - Accurate country-level prevalence data is lacking to guide programmatic actions. - The WSP has produced an M&E framework. | - Ensure robust validation and dissemination of protocols/ field manuals for standardization of mapping to ensure consistency of data. - Develop systems for tracking scabies outbreaks, monitor areas of migration and displacement camps. - Consider integrating M&E strategies with other skin diseases and with NTDs using ivermectin for MDA (onchocerciasis, lymphatic filariasis). |
| **Access and Logistics** | ◼ | - Ivermectin added to WHO Model List of Essential Medicines for ectoparasites (2019). - More manufacturers for generic ivermectin since 2019. - WHO pre-qualified ivermectin is now available in the WHO catalogue and can be procured. | - Secure supply of low-cost ivermectin and topical/systemic scabicides. - Identify further potential generic manufacturers of ivermectin that could obtain WHO prequalification. - Ensure availability of topical/ systemic scabicides and treatment at all levels. |
| **Advocacy and Funding** | ◼ | - Currently minimal donor support. - The WSP supports scabies control programs in two countries. - Limited domestic prioritization in many countries, however there is progress. | - Secure financing and ensure political commitment to support programmatic action, including the procurement of ivermectin and other scabicides. - Advocate for inclusion in universal health coverage. - Generate data on the extent and burden of scabies and the need for scale-up to underpin the advocacy and funding activities. |

### Schistosomiasis (Target: Elimination as a public health problem)

| **Dimension** | **2023**  **ranking** | **Current status** | **Recommended actions** |
| --- | --- | --- | --- |
| **Diagnostics** | ◼ | - Kato–Katz and urine filtration used to measure prevalence and intensity but suboptimal in low prevalence/intensity areas. - More sensitive and specific rapid diagnostic tests are being used and others are under development. - Published diagnostic TPPs for monitoring, evaluation and surveillance of schistosomiasis provide guidance for the development of new tests. - There is ongoing development of rapid diagnostic tests through FIND and other research groups. - Well-characterized biobanks (e.g., FIND) are consolidated and expanding. - There is a current need to develop diagnostic tests for FGS. | - Develop and introduce standardized, sensitive, point-of-care diagnostics for different prevalence/intensity settings and all schistosome species; use for mapping and transmission assessment. - Create biorepository of sera, urine and stool for diagnostic development, evaluation and validation. - Develop test for monitoring resistance to praziquantel. - Develop molecular test for xenomonitoring and surveillance, including environmental DNA. - Develop point-of-care diagnostic for female genital schistosomiasis. - Support the development and use of image analysis and artificial intelligence for automated detection of schistosomiasis. - Improve existing methods and develop new diagnostic tools to confirm interruption of transmission. |
| **Monitoring and Evaluation** | ◼ | - Working group on M&E is currently developing a new manual, including directions on how to sustain elimination as a public health problem. - There is ongoing work on granular mapping, through the Schistosomiasis Oversampling Study (SOS), and improving impact assessment surveys. | - Improve data quality and mapping to support target and track progress at the lowest level; implement granular mapping (harnessing new technologies) to support targeted MDA and other interventions at lower administrative or community levels. - Collect M&E data from pre-SAC, SAC and adults to inform optimal treatment strategy. - Improve funding to implement impact assessments for potential strategy adjustment. - Use endemicity data to target and prioritize WASH investment and track progress to elimination. - Improve reporting on disease distribution, leveraging new tools. - Implement monitoring for efficacy of and drug resistance to praziquantel. - Develop economic impact indicators to assess disease burden and programmatic progress. - Develop guidance on how to conduct post-elimination surveillance and sustain validation or verification. - Develop guidance on how to verify interruption of transmission. |
| **Access and Logistics** | ◼ | - Donation of 250 million tablets of praziquantel from Merck available for treatment of school-aged children (SAC) and some adult treatment through community delivery in the African Region. - There is a growing demand of praziquantel among programs, given the 2022 update in treatment guidelines. - Some pharmaceutical companies have been pre-qualified by WHO so that alternative sources of praziquantel are available to complement donations. - There is a lack of funding for community-based mass distribution activities. Reliance on school-based delivery of treatment can miss children not attending school, preschool-aged children (pre-SAC) and adults. - The European Medicines Agency has adopted a positive scientific position for pediatric praziquantel. Other national agencies are expected to approve this formulation. Production is likely to commence in 2024. | - Utilize donor coordination, supply, and logistic tools to ensure access to sufficient quality-assured praziquantel to treat all in need. - Ensure access to and delivery of treatment to all at-risk populations, including adults, according to the guidelines (e.g., through strengthening logistical aspects). - Ensure access to pediatric praziquantel formulation for pre-SAC, once available. - Ensure access to molluscicides. - Ensure zoonotic interventions, including praziquantel for animals, are available. - Ensure access to rapid diagnostic tests for use in the community to support test and treat activities. - Ensure and prioritize access to sanitation investments in transmission areas. - Ensure access to treatment and care for FGS. |

| **Dimension** | **2023**  **ranking** | **Current status** | **Recommended actions** |
| --- | --- | --- | --- |
| **Advocacy and Funding** | ◼ | - Currently, treatment programs rely heavily on declining external funding, which in many countries can be short-term. - Limited advocacy and fund-raising capacity at the country level. - There is limited funding available for non-treatment components of the elimination framework, including One Health and vector control, behavior change, health education, and FGS. | - Advocate to international and domestic stakeholders and policymakers to strengthen ownership of schistosomiasis control and elimination programs and their integration into universal health coverage. - Mobilize extra resources for progress towards the ultimate goal of interruption of transmission, which would allow MDA to be stopped. - Improve funding for mass drug distribution activities, including community-based distribution, to reach Pre-SAC and adults as per new guidelines. - Promote integration with other sectors to improve funding for WASH in areas endemic for schistosomiasis. Community engagement is essential for success; activities should be conducted to mobilize communities to support investments and provide long-term sustainability. - Secure funding to support the integration of control activities into the public health system, including diagnostics and treatment (e.g., treatment for women of reproductive age or FGS diagnostics) as well as reporting into M&E systems. - Develop new funding mechanisms, including domestic funding. |

### Snakebite Envenoming (Target: Control)

| **Dimension** | **2023**  **ranking** | **Current status** | **Recommended actions** |
| --- | --- | --- | --- |
| **Diagnostics** | ◼ | - Recognition of specific syndromes associated with some venoms, which helps with diagnosis. - There are no TPPs for diagnostic tools for Snakebite Envenoming. - Diagnostic tools for SE are largely unavailable for programmatic activities across the world. There is information of a single diagnostic tool being used in Australia (CSL snake venom detection kit). It is approved by the Australian Therapeutic Goods Administration (TGA) however, it is rather expensive. - Species-specific immunodiagnostics are not essential for effective treatment and are not commonly used by control programs. However, they are valuable for clinical research and disease ecology. | - Standardize and validate clinically relevant bedside diagnostic tests that confirm specific clinical syndromes (e.g., 20WBCT for coagulopathy) in specific populations. - Develop simple low-cost “Yes/No” diagnostics (immunoassay or other diagnostic methods for identification of biting species for disease ecology) to reduce delays in administration of antivenom. |
| **Monitoring and Evaluation** | ◼ | - Endemic countries conduct some M&E activities, but data collection is often limited to the requirements of the DHIS2 (District Health Information Software 2). They mostly report on cases and mortality. Reporting on morbidity is optional. - Reporting to WHO tends to vary across countries and regions. In some cases, surveillance systems might be in place, but data is not reported. - There is ongoing work to develop a more complete data system (Routine Health Information Systems, RHIS) to collect disaggregated data (e.g., sex). - Baseline epidemiological and burden of disease data are deficient, fragmented or incomplete. - There is a growing volume of epidemiological data becoming publicly available. However, data quality varies. Further research is needed to adequately validate the global burden of the disease. | - Implement mandatory reporting to improve data on disease burden. - Improve quality and extent of epidemiological surveillance (with clear common definitions of parameters) for accurate disease burden measurement and resource planning. - Develop and implement a framework to measure outcomes and outputs. - Conduct a baseline map of resources at the country level (human and products) to outline local capacity for surveillance and to manage cases. - Increase awareness of the importance of M&E activities to support programmatic activities. Funding specifically allocated to M&E activities should be mobilized. |
| **Access and Logistics** | ◼ | - Prevention activities are not commonly implemented by programs. There are recommendations for protective clothing but no formalized or institutionalized supply of products. - Antivenom provision is deficient in volume, quality, and safety. Availability is mostly observed in urban rather than in remote communities, where most at-risk people can be found. - There are significant challenges for the development of a cold supply chain system for antivenom. There might be some opportunities for integration with vaccine supply chains, but no effective collaboration mechanisms have been established yet. - There is limited evidence of the use of digital inventory management systems for antivenom. Recent developments in digital tools have centered on epidemiological data. - International donations of antivenom are limited. MoH in endemic countries commonly rely on domestic funds to acquire the product and hence are likely to cover customs clearance. However, volumes acquired are customarily insufficient. The use of domestic funds for customs clearance may further reduce countries’ purchasing capacity. - Weak confidence drives demand and supply down, raising prices and reducing availability. - Weak regulatory environments facilitate the spread of ineffective and counterfeit products. - Poor procurement, supply and distribution policies and practices and need for education and training. | - Invest in the modernization of production capacity and the expansion of overall manufacturing capacity of antivenoms and hyperimmune plasma. - Create a virtuous cycle: requires quality-assured products, market stimulus, monitoring and surveillance. - Ensure effective national and regional regulation to stop the spread of ineffective, inferior or counterfeit products. - Establish a revolving stockpile of effective antivenoms. |

| **Dimension** | **2023**  **ranking** | **Current status** | **Recommended actions** |
| --- | --- | --- | --- |
| **Advocacy and Funding** | ◼ | - Limited programmatic and national funding. - There is a general lack of international funding for SBE activities. International funding tends to prioritize research rather than programmatic activities. Not all endemic countries, however, have similar access to research funding. - There is substantive reliance on domestic funding but the countries themselves lack the resources to support all programmatic activities needed. - SE has been recently added to the NTDs list. Some countries have not yet developed dedicated control programs. - Within the NTDs funding landscape, SBE is not sufficiently prioritized. There is substantive competition with other (more established) NTDs. Lack of information on burden of disease makes it difficult to justify prioritization. - Current programmatic plans usually are short-term and do not engage with sustainability issues. - Advocacy and fundraising limited by need to identify and map donors and their interests. | - Conduct donor mapping and develop a resource mobilization strategy based on a business plan (e.g., for envenoming management) to WHO. - Mobilize domestic financing for country-level projects. |

### Soil-transmitted helminthiases (Target: Elimination as a public health problem)

| **Dimension** | **2023**  **ranking** | **Current status** | **Recommended actions** |
| --- | --- | --- | --- |
| **Diagnostics** | ◼ | - TPPs have recently been released. Kato-Katz fulfils them, as well as quality standards defined by WHO. - Progress has been made in research for novel diagnostic tools. To date, Kato-Katz remains the best option for current programmatic progress. Future programmatic actions that go beyond the current targets may include the use of other diagnostic tools. - Diagnostic methods for *S. stercoralis* have been discussed in expert meeting with report released in 2021 | - Develop rapid, more sensitive and specific, easy-to-use diagnostics for mapping and surveillance, especially with regard to assessing when STH would be eliminated as a public health problem. - Develop sensitive and specific biomarkers for field use. - Develop field tests to detect any emergence of resistance. - Standardize diagnostic procedures to minimize measurement error and to ensure comparability. - Kato-Katz needs to be available at community and facility levels, and in field settings. - Training, refresher training and quality assurance activities should be periodically conducted to maintain proficiency and ensure data quality. |
| **Monitoring and Evaluation** | ◼ | - Only some countries implement M&E to the extent indicated by current WHO guidelines. - Some countries report additional health status indicators. - Most countries adequately report on road map indicators. - Even though most countries have HMIS, only few have currently integrated STH data. - Additional global guidance on post-elimination surveillance is forthcoming imminently, however not currently available. - Countries are reporting data on road map indicators disaggregated by age and sex. Data pertaining to women of reproductive age is now included within the Joint Reporting Form. - Global guidance on disease-specific indicators is available but has not been widely integrated into HMIS. | - When applicable, utilize new cost-efficient technologies to decrease costs of mapping and surveillance. - Simplify and standardize methods for impact assessment surveys. - Drug efficacy should be monitored periodically as part of M&E activities. - Encourage countries to include data from M&E activities at program level into HMIS. - Encourage uptake of new guidelines that will be released soon. - Reporting on programmatic indicators specified in the 2030 targets for STH control programs and additional health status indicators (morbidity and mortality) should be encouraged. - Funding specifically allocated to M&E activities should be mobilized. |
| **Access and Logistics** | ◼ | - Access to mebendazole has been improved for pre-SAC, and women of reproductive age have been added as target groups. When requested, Kato-Katz diagnostic kits are available through WHO free of charge. - Medicines for STH are available for individual patient care. - Supply chain organization is efficiently managed by most ministries of health. - The majority of countries have a digital inventory system at the national level but not at local levels. - Customs clearance does not need to be covered by domestic funds as it is included in drug donation. For in-country delivery, existing infrastructure and supply mechanisms are used, which is adequately covered by domestic resources. - Ivermectin is now available as pre-qualified generic medicine, however donations remain to be secured. | - Continue to improve access to medicines for women of reproductive age and pre-SAC. - Increase the availability of ivermectin in *T. trichiura*-endemic areas, when indicated. - Ensure in-country distribution and supply chain of products are able to reach all health care levels, including the periphery. - Disseminate knowledge about products available through WHO |

| **Dimension** | **2023**  **ranking** | **Current status** | **Recommended actions** |
| --- | --- | --- | --- |
| **Advocacy and Funding** | ◼ | - Most countries have an NTD master plan (or equivalent) which includes STH within an integrated strategy. - The majority of countries have international funding for NTDs, with either dedicated funding for STH, or in which STH control can be included. - Even though currently the majority of countries are able to support programmatic actions by tapping into personnel and domestic resources, they heavily rely on funding coming from international sources. - Since most funding comes from international sources, only some endemic countries have a dedicated budget line for NTDs. - Most effective advocacy is done by international organizations and does not come from the countries themselves. This may be due to most programs being part of governmental entities and are thus restricted in terms of advocacy for funding. - In practice, only some endemic countries have a sustainability plan, even though the majority mentions it in theory within the NTD master plan. - Many countries remain dependent on drug donations and external funding for program implementation. - The number of donated tablets needed is expected to decrease substantially as countries become self-sufficient and as the frequency of preventive chemotherapy decreases after successful interventions. - No funds or donations are currently available for control of strongyloidiasis. | - Increase domestic financing to ensure sustainability in a changing international funding landscape. - Secure drug donations for women of reproductive age and preschool-aged children. - Advocate for expanded water access, and sanitation and waste management, in endemic areas. |

### Taeniasis and Cysticercosis (Target: Control)

| **Dimension** | **2023**  **ranking** | **Current status** | **Recommended Actions** |
| --- | --- | --- | --- |
| **Diagnostics** | ◼ | - Practical diagnostic tools for taeniasis, such as fecal exams, are not sensitive enough; sensitive tools are too expensive. - Diagnostic tools for porcine cysticercosis are not specific and sensitive enough. - Molecular tests for species identification are being validated for Taeniasis. - POC test prototype for neurocysticercosis has been developed which may support *Taenia solium* cysticercosis and taeniasis control programs in the future. | - Develop and validate specific and sensitive diagnostic tools which are practical and affordable for porcine cysticercosis. - Devise a sensitive and specific test that is practical and affordable for taeniasis. - Improve and validate the POC test prototype for active neurocysticercosis. |
| **Monitoring and Evaluation** | ◼ | - WHO M&E framework is under development and due to be released in 2024. - M&E systems do not exist in most endemic countries. | - Conduct mapping at sub-national level in endemic countries to fully understand the scope of the challenge and disease burden. Integrate if possible with other programs such as schistosomiasis and soil-transmitted helminths. - Devise appropriate diagnostic tools to accurately assess and map endemicity, to inform control programs for taeniasis and asymptomatic NCC in resource-limited settings. - Integrate M&E strategies and improve data collection across sectors at country level to include animal, human, and environmental health following the One Health approach. - Harmonize data collection and reporting to coordinating authorities including WOAH and WHO; improve surveillance, data quality and digital data management at country level. |
| **Access and Logistics** | ◼ | - Limited availability of praziquantel in the market. - Existing donation of praziquantel and niclosamide are from Bayer, with ongoing discussions to increase amounts. - Efforts are geared towards improving access to antiepileptic medications and anthelminthics for neurocysticercosis (NCC) patient care, in collaboration with mental health sector. - Pig vaccination is now incorporated in the WOAH Terrestrial Code and Manual, but there is little demand due to low prioritization of pig vaccination in the animal sector, which hinders the amount of stock commercial vaccine production from the sole manufacturer. - There is currently no donation of oxfendazole and vaccines for treatment / vaccination of pigs due to the limited morbidity caused in pigs. Therefore, farmers have to purchase treatment and vaccinations for pigs out of pocket. | - Register TSOL18 pig vaccine and oxfendazole for treatment of porcine cysticercosis in endemic countries and integrate them into existing distribution and veterinary systems to ensure their availability. - Increase access to anti-seizure medications required for NCC patients and other epileptic patients in primary health care and beyond. - Increase availability and accessibility of NCC diagnostics, including CT scans and neural MR imaging, especially in rural areas. - Increase the access to anthelmintics for NCC treatment. - Improve primary healthcare workers' knowledge of the indications for NCC treatment, mainly when patients should receive anthelminthics or be referred to hospital. - Advocate for the inclusion of treatment of NCC in governments’ essential medicine lists and increase government prioritization. - Advocate for the implementation of a One Health approach focusing on control in people (including patient management) and pigs. |
| **Advocacy and Funding** | ◼ | - Advocacy from countries and stakeholders is increasing, but the response from governments and philanthropic agencies to invest in control is lacking. - Control activities in animals lack prioritization, the impact lies primarily within non-commercial, smaller-scale backyard pig farming that do not understand the disease and cannot afford control measures. | - Encourage advocacy from WHO/FAO/WOAH/UNEP through a One Health lens for disease management and control activities. - Dedicate a One Health budget line in endemic countries to fund and support animal health and other NTDs. - Increase commitment of governments and partners to prioritize taeniasis/ cysticercosis through evidence collection on disease burden and economic impacts. |

### Trachoma (Target: Elimination as a public health problem)

| **Dimension** | **2023**  **ranking** | **Current status** | **Recommended Actions** |
| --- | --- | --- | --- |
| **Diagnostics** | ◼ | - Undertaken based on clinical examination by trained clinical graders. | - Conduct research to understand whether tests for current or previous ocular *C. trachomatis* infection would help programs determine the need for interventions or monitoring populations, after interventions are discontinued (i.e., between impact and surveillance surveys). If so, consider developing serological rapid diagnostic tests to support elimination and post-elimination surveillance. |
| **Monitoring and Evaluation** | ◼ | - The Global Trachoma Mapping Project was successfully completed, with limited areas remaining unmapped by 2016. Trachoma mapping is yet to be completed but there has been significant progress through Tropical Data, which has identified unmapped areas for trachoma. - WHO Global Health Observatory and the GET2020 database provide a global data repository. | - Complete remaining limited mapping. - Develop systems to track surgeries and outcomes. - Devise methods for improved and sustainable surveillance and post-validation surveillance to limit recrudescence of infection. - Design joint (inter-sectoral) M&E indicators as appropriate (e.g., WASH) - Develop a better indicator of TT to determine elimination as a public health problem and a better way to estimate the backlog of TT cases. |
| **Access and Logistics** | ◼ | - Pfizer has donated more than one billion doses of azithromycin since 1992 through the International Trachoma Initiative. Pfizer continues to support donations despite current production challenges. - National systems manage supply chain with help of partners. | - Continue to maintain, review and improve systems as needed. - Maintain support to prevention and management of serious adverse events. - Continue to work with donors to overcome current azithromycin shortages. |
| **Advocacy and Funding** | ◼ | - Countries are providing critical core funding, usually covering staff costs for trachoma initiatives. - Interventions against trachoma have been identified as a “best buy” and donors include both bilateral and private donors. - Program operations are significantly reliant on international funding, but efforts are taking place to seek additional domestic funding. | - Mobilize domestic and other financial resources and political will to implement SAFE at scale and sustain elimination. - Develop return on investment case priorities for F&E and WASH as evidence is generated. - Programs need to continue working on increasing domestic funding. |

### Tungiasis (Target: Control)

| **Dimension** | **2023**  **ranking** | **Current status** | **Recommended Actions** |
| --- | --- | --- | --- |
| **Diagnostics** | ◼ | - There are no current defined program use-cases for diagnostics. - Methods exist for screening and individual diagnosis. | - Establish the accuracy of current diagnostic methods, including clinical examination. - Develop programmatic uses cases accompanying WHO TPPs and guidelines for diagnostics. |
| **Monitoring and Evaluation** | ◼ | - Burden of disease and risk factors are poorly understood. - The transmission dynamics and role of zoonotic reservoirs of the disease are insufficiently understood to inform M&E frameworks. - No M&E framework or data systems established for this disease. | - Design operationally feasible mapping and tracking strategies. - Develop and disseminate protocols for standardization of mapping to ensure consistency of data. - Consider integrating M&E strategies with other skin diseases and a One Health approach. - Promote inclusion of Tungiasis in national HMIS for reporting and monitoring. |
| **Access and Logistics** | ◼ | - No reliable supply chain for preventative interventions or individual patient care. | - Ensure availability of dimeticone in all endemic countries. - Ensure the availability of treatment for animals. - Secure access to treatment for secondary infections and treatment complications. |
| **Advocacy and Funding** | ◼ | - Currently minimal donor support and limited domestic prioritization in almost all countries. - Very limited visibility of Tungiasis in the current public health and research agenda. | - Create an advocacy and funding plan. - Increase advocacy for operational research for Tungiasis to generate more evidence to enhance control efforts and gain donor support. - Secure financing for topical treatments. - Advocate for inclusion in universal health coverage and One Health approach. - Integrate interventions for Tungiasis, alongside other skin NTDs. |

### Yaws (Target: Eradication)

| **Dimension** | **2023**  **ranking** | **Current status** | **Recommended Actions** |
| --- | --- | --- | --- |
| **Diagnostics** | ◼ | - Point-of-care serological assays can be used for screening and confirmation. - Molecular diagnostics are available for confirmatory testing and drug resistance monitoring. - LAMP is being investigated for field-based point-of-care application. - Access to diagnostics remains a challenge. - TPPs were published in 2022. | - Develop a sensitive and specific point-of-care test (for example PCR) to distinguish yaws from other skin ulcers/lesions. - Develop a point-of-care test to distinguish asymptomatic yaws cases from other treponemal infections (e.g. syphilis). |
| **Monitoring and Evaluation** | ◼ | - Surveillance systems are well established in some endemic countries, however, not to scale. - WHO M&E framework for yaws published and strategic framework for skin NTDs released. - Implementation of M&E activities is weak due to a lack of diagnostic tests and funding for implementation. | - Establish active integrated surveillance in all yaws-endemic and formerly endemic countries (status unknown). - Increase the frequency of country reporting on M&E indicators to monthly. - Encourage and provide technical support to formerly yaws-endemic countries and territories to assess the current status of yaws. - Increase access to PCR for disease confirmation and to monitor antimicrobial resistance. - Include disabilities due to yaws as additional health status indicators. |
| **Access and Logistics** | ◼ | - Availability of azithromycin for MDA is covered by donations, however access is not ensured. - Limited availability of antibiotics and diagnostics within primary health care facilities to diagnose and treat cases and contacts (TTT). - In remote areas, access to medicines and diagnostic tools (e.g. RDTs/DPPs) is difficult. - Some countries have azithromycin in national essential medicines list, however not for the treatment of yaws. | - Improve access to diagnostic tests and medication in all endemic locations, including isolated foci (as part of universal health coverage). - Ensure access to point of care tests and sufficient quantities of medicines for MDA and TTT that are of assured quality. - Ensure that yaws is included as an indication for azithromycin on national essential medicines list. |
| **Advocacy and Funding** | ◼ | - Limited political and donor/partner support for implementation. - There is buy-in of communities to MDA and awareness among endemic population is improving. - There is some advocacy support from the research community. | - Increase funding and advocacy for yaws eradication, including securing longer-term commitments and increasing the priority of yaws as suitable for preventive chemotherapy and as a skin NTD. - Sustain community engagement to support program implementation. - Maintain research community engagement for knowledge generation and advocacy to mobilize resources for research. - Mobilize funds for conducting programmatic activities in addition to medicines and diagnostics delivery. - Adequate resources are needed for yaws eradication, including diagnostics supply and funding of activities and personnel. |
