# Supplementary material for: Tracking progress along the WHO Neglected Tropical Diseases Road Map to 2030: A guide to the Gap Assessment Tool (GAT) and results from the 2023–2024 assessment
Source: PLoS Negl Trop Dis. 2025 Jul 1;19(7):e0013194. doi: 10.1371/journal.pntd.0013194 (PMC12244624; doi:10.1371/journal.pntd.0013194)
Supplement: S3 Table — Recommendations from the dimension-level focus group discussions are presented, for each of the four dimensions. (DOCX) [file pntd.0013194.s003.docx]

| WHO-GAT DIMENSION-LEVEL (CROSS-CUTTING) RECOMMENDATIONS (Dimensions: Diagnostics, Monitoring and Evaluation, Access and Logistics, and Advocacy and Funding) |
| --- |

## Diagnostics

| 1. **Development of integrated diagnostic tools for multiple NTDs** |
| --- |
| - Encourage the **identification and validation of biomarkers for multiple NTDs**, across several countries, with the aim of developing multiplex tests, preferably using the same sample type and platform. The composition of multiplex tests needs to be aligned to the geographical distribution of NTDs across regions and be validated according to their specific requirements. - Ensure the **development of multiplex tests is aligned with relevant use-cases** **shared by NTDs, as outlined in their respective Target Product Profiles** (TPPs). Ongoing work in the Skin-NTDs sector may inform future initiatives of this kind. - Investments in the development of multiplex tests should aim to **produce rapid diagnostic tools that can be deployed in primary care services** (i.e., point-of-care (POC) tests). - Ensure that **investments in the** **development of integrated tests accompany, rather than supersede, efforts to develop simple single disease kits** for key NTDs**.** Ambitious targets (e.g., development of comprehensive multiplex rapid diagnostic tests (RDTs) for POC) may involve extended timelines. Programmes’ current diagnostic needs and priorities should be taken into account. - **Develop suitable programmatic guidance on integrated diagnostics to accompany their release** so that programme managers can effectively, and flexibly, adapt the new tests to their specific epidemiological and programmatic requirements. - **Promote cross-cutting agreements on technical profiles for multiplex tests**, concerning the minimum and quality performance characteristics as well as operational features required to address the specific programmatic needs of all relevant NTD programmes concerned (e.g., TPPs). |
| 1. **Development of integrated diagnostic tools for zoonotic NTDs** |
| - Encourage **coordination across programmes linked to zoonotic NTDs** **to support the development of integrated diagnostics** (e.g., trematodiases, cysticercosis, cystic echinococcosis)**.** - Ensure the **development of integrated diagnostics for zoonotic NTDs (simultaneously work on producing tests for both humans and animals)** given the central role played by animals in the transmission cycle. - **Programmatic guidance should be produced for the use of integrated tests in control and elimination activities for zoonotic NTDs**. Emphasis should be placed on diagnostic activities aimed simultaneously at humans and animals. |
| 1. **Development of integrated tests involving non-NTD diseases** |
| - **Examine the potential of existing tests for non-NTD diseases** **that may serve as a platform for integration with NTDs.** Established tests for non-NTDs may provide cost-effective and time-efficient opportunities for the development of integrated tests (e.g., skin diseases not listed as NTDs). - **Ensure that the costs of tests integrated with non-NTD platforms are affordable to relevant programmes.** Existing tests for non-NTDs may have established manufacturing and procurement conditions that can be affected if integration results in a significant increase in manufacturing and distribution costs. Efforts should be made to support the uptake of integrated diagnostics, once available, to help drive costs down over time, as usage increases. |

| 1. **Advocacy to secure support for integrated diagnostics** |
| --- |
| - Coordinated advocacy efforts should be conducted to **expand donor support for the development of integrated tests through multiple platforms** (e.g., serological or molecular). Advocates should strive to ensure donors adopt a more holistic approach to tackling NTDs. - Coordinated advocacy efforts should be conducted to **widen donor support for the development of tests for zoonotic NTDs and diagnostic activities concerning animal health**. Programmes should strive to promote a One Health approach among donors so that funding for diagnostic activities adequately budget for human and animal components. - Compile and disseminate evidence concerning the **health and economic benefits of integrated approaches to diagnostics development and activities,** which can strengthen advocacy activities. Information on the economic value of integrated activities can facilitate inter-sectoral coordination with veterinary and agricultural sectors, given the productive role of some animals. - **Engage with global stakeholders and manufacturers to secure access to multiple suppliers** in order to ensure long-term production of emerging integrated tests. |
| **5. Mainstream diagnostic activities within the public health system** |
| - **Leverage existing public health services to enhance diagnostic capacity for relevant NTDs** (e.g., female genital schistosomiasis and cervical cancer screening). - **Seek integration with public health systems’ initiatives aimed at strengthening laboratory services and capacity at the primary care level.** This can enhance programmes’ delivery and coverage of diagnostic activities, including in remote settings. - **NTD programmes should engage with initiatives to expand and strengthen national laboratory services and systems within public health systems** in various countries. This could enhance their diagnostic capacity through greater access to qualified staff, training, and equipment to support integrated activities. - **Conduct a country-based assessment of** **public health systems** to ascertain which services that are commonly offered to the public across countries offer the most effective pathways and mechanisms for integration with NTDs’ diagnostics activities. - **Develop programmatic guidance on integration approaches with public health systems according to NTD programmes’ road map objectives.** Integration approaches are likely to differ depending on the type of diagnostics operations required to reach targets (e.g., longer term requirements for efforts focused on elimination as a public health problem, compared to elimination as interruption of transmission). |
| **6. Development of regional (supra-national) laboratory capacity.** |
| - **Support the development of a regional supra-national reference laboratory for NTDs in each WHO region.** These could provide (i) surge capacity in case of outbreaks (ii) training workshops to national and regional laboratories (iii) reference facilities for uncommon or experimental testing and (iii) quality assurance direction, ensuring that national laboratories utilise adequate and comparable methodologies, tools, and processes, keeping them up to date with any recent developments. - **Advocate to WHO to support the development of a supra-national reference laboratory network for NTDs.** WHO collaborating centers could be useful as an initial network to develop such centers (e.g., Skin NTDs Laboratory Network, WHO AFRO; ESPEN Laboratory; or Zanzibar’s Public Health Laboratory). |
| - **Encourage NTD programmes to participate in existing initiatives aiming to establish regional supra-national bodies for strengthening and expanding diagnostic capacity and networks** for emergency, communicable, and pandemic diseases, among others (e.g., African Centre for Integrated Laboratory Training, Regional Integrated Surveillance and Laboratory Network). This can enhance NTD programmes’ laboratory capacity and surveillance systems. Strategic and programmatic guidance, however, is still required to outline effective mechanisms for integration between NTD programmes and such initiatives. - **Advocate to WHO to facilitate the gathering and transferring of specimens/samples for NTDs across countries**, which current regulations render difficult. There is a need to establish large repositories to develop and validate new tests. This is problematic for less frequent NTD infections since it is not easy to gather the required large quantity of samples. |
| **7. Inter-sectoral collaboration for enhanced diagnostic capacity and development.** |
| - NTDs programems could benefit from collaborating and coordinating with **Water and Sanitation and the Environment** sectors. This would allow programmes to triangulate data (e.g., through environmental sampling) to support mapping and planning for elimination as well as to coordinate vector control activities. - Coordinating and integrating activities with **Veterinary and Agriculture** sectors could benefit NTDs programmes for zoonotic diseases in three areas: (i) improved access to laboratories, widening their diagnostics capacity for vector control and surveillance; (ii) ability to triangulate data to support mapping and planning for elimination as well as coordinate vector control activities; and (iii) access additional resources (materials or information) to support the development of new or improved tests for humans and animals. - **Develop programmatic guidance for inter-sectoral collaboration on diagnostics, under a One Health approach**. There is potential for high impact from implementing simultaneous operations across sectors (e.g., environmental, veterinary, and human diagnostics in targeted areas). Lessons can be derived from the echinococcosis programme in Argentina, where screening and treatment for humans happened alongside animal vaccination and deworming. Relevant funding support should be sought. - **Support the establishment of coordinating offices for inter-sectoral collaboration, within Ministries of Health.** This will ensure formal roles and responsibilities within the existing administrative structures to advocate and plan for inter-sectoral collaborative initiatives that can enhance NTD programmes’ diagnostic activities. - **Develop collaborative initiatives with the Education sector** to strengthen higher education programmes for health professionals and widen their offer of technical workshops for practitioners. These could support programmes’ diagnostics activities by ensuring appropriate in-country technical capacity across all levels. |

## Monitoring and Evaluation

| 1. **Implementation of integrated M&E activities across NTDs, with the public health system, and non-health sectors** |
| --- |
| - **Encourage the implementation of integrated M&E activities and tools across NTDs**, for activities such as mapping, impact assessments, or drug efficacy monitoring. NTDs with similarities in transmission, geographical distribution, treatment strategies, drug-delivery platforms or M&E approaches can benefit from sharing resources, as a cost-saving strategy**.** - **Encourage the implementation of integrated M&E activities between NTD programmes and public health services** with compatible platforms (e.g., female genital schistosomiasis with cervical screening, sexual and reproductive services; immunization services, especially community-based or outreach programmes). This could widen programmes’ access to material and human resources and coverage among target populations, enhancing their efficiency. - **Support integrated surveillance activities among NTDs that are yet to reach elimination and relevant non-NTD diseases** (e.g., those with compatible transmission vectors or treatment platforms). This could allow programmes to ensure access to the resources necessary to support their M&E operations in the long-term. - **Coordinate with the Veterinary and Agriculture sectors to harmonise M&E activities** under a One Health approach, where appropriate. M&E information on progress towards road map targets can be enhanced by collecting matching data for both populations. |
| 1. **Development of integrated digital M&E systems and tools** |
| - **Encourage the use and continuous refinement of the Global NTD Annual Reporting Form (GNARF),** which provides an integrated platform for reporting essential M&E data, progress, and activities across multiple NTDs within a given country. - **Support the development of integrated information platforms that enable M&E information sharing between NTD programmes and public health services**, building up on existing Health Management Information Systems (HMIS) or similar reporting systems. This could generate feedback mechanisms to plan NTD interventions and public health responses (e.g., FGS with sexual and reproductive health services or snakebite with emergency services). Programmes can build on or expand existing regional or country initiatives, such as the Integrated Disease Surveillance strategy launched by EMRO to generate national surveillance capacities using an electronic platform. - **Develop or expand existing integrated global data platforms for M&E** among those NTDs sharing similar control and elimination strategies and M&E approaches. Access to up-to-date M&E data, disaggregated and cutting across all levels of operations, can help planning activities within and across countries. The ESPEN initiative may serve as a reference to inform other similar initiatives. - **Encourage the use of Artificial Intelligence tools to improve M&E data and reporting systems**. Efficacy can be improved through automated data harmonization and integration as well as reporting. Efforts should be made to assess the potential of existing tools to support this work. - **Secure technical support for capacity building within countries** to support the development and efficient use of integrated information and reporting systems. |
| 1. **Data policies for integration and mainstreaming of M&E systems** |
| - **Advocate to WHO and partners supporting NTD programmes (e.g., donors, iNGOs) to harmonize their M&E information requirements,** so that data collection activities can be streamlined and reporting systems easier to integrate. - **Establish information agreements between NTD programmes and relevant public health programmes concerning** **indicators, data collection approaches, and information sharing mechanisms.** Ensuring data compatibility between M&E systems across the health sector is essential to facilitate integration between platforms, both at the national and global levels. - **Ensure that NTD programmes and public health services are informed of data protection policies relevant to their respective M&E systems and coordinate the adoption of suitable data sharing policies.** Support should be provided to NTD programmes to assess and adapt legal restrictions in their efforts for integration. - **Establish M&E data-sharing mechanisms for cross-border disease transmission information**, with particular emphasis on NTDs targeting elimination and eradication. Coordination through WHO regional offices might facilitate this process. - **Establish M&E data-sharing mechanisms across programmes working with mobile populations** (e.g., migrants and nomadic groups), **including those from the humanitarian sector** (e.g., refugees and internally displaced populations). Coordination through WHO and other relevant UN agencies (e.g., UNHCR) will be necessary to facilitate this process. - **Coordinate with other sectors, including veterinary, WASH, and agriculture to seek harmoniastion in relevant M&E indicators and reporting systems,** to facilitate information exchanges and data triangulation. Ensure that proposed data collection strategies and indicators are representative of NTD programmes’ target populations. - **Identify or develop indicators within NTD programmes’ M&E systems that can feedback into other sectors**, such as WASH or animal health. Efforts to specify how NTD programmes can contribute to other sectors’ evaluation activities can facilitate inter-sectoral coordination and the establishment of formal communication channels. |
| 1. **Operational research to support M&E integration** |
| - **Conduct a cross-country review of programmes’ experience with the Global NTD Annual Reporting Form (GNARF) system.** This activity could serve to assess existing needs on data compatibility, user experience, training requirements, time-labor demands, as well as the suitability of included indicators. - **Support operational research initiatives to provide evidence on the value of using integrated M&E information systems to plan for programmatic activities**, examining their impact on the standardisation of data collection approaches, data quality, and efficiency gains. This can be valuable for advocacy purposes, in addition to supporting planning for similar initiatives by other programmes. Lessons may be drawn from ongoing projects, like ESPEN and ESPEN collect. - **Conduct a sector-level assessment of progress and challenges in adopting and implementing M&E guidelines and frameworks.** This will serve to pinpoint specific M&E operations where integrated approaches may generate maximum impact as well as to gather information on existing integration initiatives. |
| 1. **Development of technical guidelines across NTDs** |
| - **Encourage close coordination between ongoing M&E initiatives to produce post-elimination surveillance guidance and those aiming to develop integrated diagnostic tests**, including multiplex**.** Technical developments in integrated diagnostic tools should be adapted to the practical requirements of conducting M&E under post-elimination scenarios, since methodological requirements and approaches are likely to be dissimilar across diseases. - **Complete the development of standardised guidance for the acknowledgment processes across NTDs.** Specific decision-making mechanisms and processes are needed across the sector to consistently advise different programmes when they can move from one stage to another, from control to eradication. Methodological requirements proposed need to consider countries’ different epidemiological trajectories, scenarios, and public health priorities, given the costs involved. - **Develop technical guidance for the design and implementation of M&E activities in fragile, conflict-affected and vulnerable (FCV) settings.** NTD programmes could benefit from accessing sector-level guidance on how to ensure the continuity of essential M&E operations in the context of humanitarian emergencies. - **Develop or update basic M&E guidance for NTDs lacking suitable diagnostic tests** to support field operations**.** A minimum set of standards and methodologies should be established across those programmes to produce consistent reports on progress and impact of programmatic activities. |

## Access and Logistics

| 1. **Information systems to improve forecasting across NTD programmes** | |
| --- | --- |
| - **Improve access to good quality health and population data for forecasting for all NTDs**. These are essential to assess disease burden and estimate the health product demands for manufacturers and stakeholders, including funders. Such estimates are likewise critical to inform advocacy initiatives to raise additional support, when required. - **Adapt existing standardised forecasting tools used by NTD and non-NTD programmes to support programmes lacking such tools.** Some programmes lack sufficient quality data and technical support to conduct adequate forecasting. Relying on tested tools can streamline this process (e.g., health indicator data, morbidity-based forecasting, or epidemiological data). Learning from programmes like tuberculosis, malaria or leprosy may be informative. - **Conduct a sector-level assessment of previous forecasting activities to examine their accuracy and efficiency.** A review of past forecasting activities is necessary to identify areas for improvement, harmonise methods across compatible NTDs (e.g., sharing similar treatment strategies), and seek strategies to integrate operations into the national health system. - **A single platform for NTD supply chain management and forecasting needs to be developed for country reporting and requests**. This would ensure harmonisation of methodologies and indicators across NTDs as well as regions, in addition to facilitating coordination for advocacy purposes across NTDs sharing similar needs (e.g., drug requests or target populations). - **Establish feedback and coordination mechanisms between programmatic areas to inform forecasting for NTDs.** Access and logistic systems require access to up-to-date monitoring and evaluation as well as financial information from their programmes as well as from others, when integration is pursued. Existing information platforms should establish standard feedback mechanisms between key programmatic areas across the sector. - **Develop a clearly defined set of minimum required indicators for reporting on logistics across NTD programmes.** Global agreements across stakeholders, including donors, concerning the definition and adoption of common indicators can streamline and harmonise reporting, ensuring greater consistency across different projects and programmes. Standardisation of indicators across the sector can also facilitate integration of NTD logistics reporting into national LMIS. | |
| 1. **Support integration of management and logistics into the national health system** |  |
| - **Encourage a greater integration of distribution and delivery procedures for NTD products into the national health system.** This strategy can streamline operations, increase programmes’ coverage, and save costs, through access to national distribution centers and local warehouses, for example. - **Integrate NTD management and logistics operations into existing country management information systems, either LMIS or HMIS, used by the national health system.** This measure can help to avoid costly duplication of efforts. In addition, donations and health products across the health sector could then be more efficiently managed through the national health system. |  |
| 1. **Advocacy to support access and logistic systems** | |
| - **Advocate at the global and country levels to enhance resource mobilisation for access and logistics systems across NTD programmes.** Enhanced support is required to improve existing systems and ensure capacity to respond to changes in disease incidence and prevalence. Focusing discussions on current levels of need (global demand), as compared to actual funded demands, could support advocacy activities across all levels (global to country). The presence of NTD programme representatives in the forecasting and planning activities within the national health system may further improve country-level support. - **Advocate to donors for their continued support in post-elimination settings to ensure an adequate supply of health products, including diagnostics.** Donations often decrease significantly after elimination validation, which can jeopardise sustaining achieved gains. Donations of drugs and diagnostics are still needed for treatment in remaining hotspots and prevent re-emergence of diseases in case of outbreaks. - **Advocate to governments that NTD programmes should be fully exempt from paying taxes over the importation of drugs and other health products.** Reducing such payments can increase programmes’ purchasing capacity or enhance the financial resources available to support other activities. - **Advocate to NTD programmes on the benefits of integration for access and logistics.** Currently, programmes often follow a vertical approach, planning their operations in a siloed manner. Limited understanding of the benefits of integration, across NTDs and with the public health system, hinders collaboration and the development of streamlining initiatives. - **Ensure greater participation of access and logistics experts in the development of programmatic guidance across the sector.** Implementation and technical guidance for programmatic activities are often developed with limited input from logistics teams, concerning feasibility and costs associated to new recommendations (e.g., updated treatment or M&E guidelines). This can generate new and sudden strains on existing supply chains and delivery systems. - **Conduct advocacy at the community level to support the development of demand-based estimates of programmatic needs.** Due to stigma associated to some NTDs, affected populations’ demand for treatment and interventions can be low, affecting programmes’ capacity to adequately establish the level of support required for their field operations. | |
| 1. **Strengthen supply and procurement of NTD medicines and health products** |  |
| - **Coordinate with stakeholders across the NTD sector to secure global availability of required health products**. Due to the humanitarian nature of NTD programmes, there are limited financial incentives for manufacturers**.** Some programmes, chiefly relatively small ones targeting pockets of disease, are at risk of losing their suppliers over time. Sector-level coordination with manufacturers, donors, and multilateral organizations is needed to expand the network of suppliers for key products or maintain existing ones. - **Encourage NTD programmes to develop forecasting estimates beyond one-year cycles.** Producers and donors would be in a better position to ensure an adequate supply of health products if programmes estimate their needs for extended periods of time. This could enable them to have sufficient production capacity to cope with demand fluctuations and avoid stockouts. - **Establish standard requirements for medicines and health products procured outside donation schemes in alignment with programmes’ needs.** Countries procuring their own health products often rely on country-based quality standards guidelines, which may not follow similar standards to the WHO initiatives. Lack of options in the international market can further affect access to quality products. Pre-qualification of treatments and diagnostics products should be further strengthened to ensure access to products of good quality standards, in sufficient quantities over time. |  |
| 1. **Inter-sectoral coordination for zoonotic NTDs** |  |
| - **Support the integration of field operations for zoonotic diseases between NTDs and relevant sectors, like veterinary or agriculture, as part of a One Health strategy.** Coordinated action to intervene humans and animals simultaneously can widen programmes’ access to resources and enhance the impact of treatment activities. - **Foster advocacy initiatives for zoonotic diseases integrating NTDs and relevant sectors, like veterinary and agriculture, to secure adequate access to treatments for animals and humans.** NTD programmes operating in low resource settings, particularly if targeting small pockets of disease, have limited chance to acquire sufficient products to achieve their road map objectives. Tri-partite coordination across global stakeholders, like WHO, FAO and WOAH, can engage with manufacturers and donors to support integrated interventions in a sustained manner. At the country level, relevant NTDs can seek being included into national plans for zoonotic diseases. |  |

## Advocacy and Funding

| 1. **Development of sector level guidance for robust and effective advocacy across NTDs** |
| --- |
| - **Produce a disease-level assessment that identifies shared priorities and bottlenecks to guide the integration of advocacy initiatives across multiple programmes.** NTD programmes have their respective public health targets, intervention strategies, and funding needs. The integration of advocacy activities across disease programmes should be strategic, aligned to specific common needs and opportunities for collaboration, to ensure their effectiveness. Advocacy integration can be focused on key themes (e.g., school health) and programmatic areas (e.g., M&E), according to how they fit into programmes’ different agendas. - **Conduct a landscape assessment of advocacy and funding priorities across national, regional, and global levels to guide multi-level coordination and integration efforts.** National advocacy agendas can differ across countries, given their different circumstances (e.g., prevalence of NTDs or public infrastructure), and from campaigns at regional and global levels, which respond to other considerations (e.g., political landscape). To maximize their impact, advocacy initiatives should be aligned around complementary priorities and stakeholders across different levels of operations. High-level advocacy can promote overarching strategic themes to mobilise funders, which can be linked up with national-level stakeholders engaged with the implementation of associated activities. - **Examine key stakeholders’ priorities and processes across all levels, from local to global, to inform the development of advocacy plans**. The development of advocacy plans should proactively seek funding opportunities and strategically select where to focus their efforts to maximise their chances of success. This may demand gathering information on the priorities of institutional funders (within and beyond the NTDs sector), including their decision-making processes for funding assignation, as well as on key national or regional policy makers with the capacity to influence funders’ agendas. A good understanding of funders’ operations and commitments can contribute to identifying hidden gaps and opportunities for NTD funding. - **Develop operational guidance on the design and implementation of advocacy campaigns promoting integrated initiatives across NTDs and with the public health sector.** Various programmes have already managed to gather support for the implementation of integrated initiatives. Lessons and best practices from those past experiences can be systematically gathered, through case studies, to develop operational guidance outlining key processes and strategies for the design and implementation of similar advocacy initiatives. Accounts from key stakeholders, including managers, front-line workers, and end-beneficiaries can provide solid evidence to inform such guidance. Regional meetings of programme managers could serve as a platform for related discussions, for reporting on progress for existing initiatives, and inform on strategies used to address different epidemiological and institutional contexts. - **Encourage programmes to outline plans to address likely future scenarios for NTDs.** Advocacy plans could be outlined in response to likely future challenges and requirements, drawing on relevant landscape assessments and strategic planning exercises, so that early measures can be adopted to ensure long term sustainability. Measures may include supporting integrated activities between NTDs with similar epidemiological trends (e.g., transitioning from elimination to eradication), south-south partnerships to address emerging challenges (e.g., cross-country transmission or scale up local production of health products), and intersectoral collaboration aligned with global health priorities (e.g., climate change). |
| 1. **Strengthened programmatic structures supporting advocacy efforts** |
| - **Strengthen reporting mechanisms on funding and expenditure indicators for NTDs.** Financial contributions are not often reported in a standardised and up-to-date manner, chiefly domestic funding. WHO’s system on health expenditure (Global Health Expenditure Database) collects information on government expenditure and external funding per country but quality and frequency of reporting varies. Technical support is required to improve financial estimates and reporting as well as to ensure consistency across countries and programs. Reporting systems may also benefit from further disaggregation, to enable comparisons between diseases, and greater dissemination (e.g., Kigali Declaration Commitment Tracker). These actions can further enhance advocacy efforts by showcasing greater transparency, helping to develop cost-benefit analyses, and demonstrating existing political will and commitment to support NTD programmes. - **Secure support for region-level advocacy efforts fostering data sharing and cross-country coordination for programmatic activities across multiple NTDs.** Material and institutional support should be provided to regional collaborative platforms that provide access to relevant up-to-date epidemiological data or help coordinating targeted interventions to address the risk of cross-border transmission. Cross-regional advocacy initiatives may likewise be considered, to support information and learning exchanges as well as to standardise frameworks. High-level, regional or global, advocacy can overcome political differences preventing cross-country collaboration, build political support, and encourage engagement from national programmes. - **Reinforce WHO headquarters’ support to, and engagement with, advocacy initiatives organised by regional offices.**  Combined efforts between WHO regional offices and headquarters could have a significant impact in securing access to domestic (government) funding as well as mobilising new international partners to support regions lagging behind in access to funding. Strengthened WHO-HQ support for key regional bodies, like ESPEN, can enhance access to more targeted, subject-specific, support for national programmes. High-level advocacy, however, requires engagement and input from countries to ensure adequate local buy-in and support. This would include information from other departments/areas within the programmes themselves not typically consulted in the past, as well as from experts specifically consulted to address recognised challenges or weaknesses in programme implementation. Access to joint funding opportunities may be helpful to this effect. - **Build communications capacity across NTD programmes.** National programmes could benefit from having access to technical support on communications, either through regional or global initiatives, that could build local capacity for the design and implementation of advocacy campaigns tailored to different audiences, ranging from community stakeholders to government officials. Ensuring access to marketing expertise could be beneficial to outline new and more effective advocacy messages and strategies. - **Encourage programmes to monitor and take advantage of epidemiological trends in different regions to expand their advocacy activities and reach new stakeholders.** Some NTDs are entering new regions and countries, including the Global North (e.g., dengue, leishmaniasis). While this circumstance may add challenges to programmes, it can serve to attract new partners and funders, potentially providing new momentum to advocacy activities supporting associated control and elimination interventions. - **Encourage programmes to outline their advocacy plans within their respective national master plans.** Adding a specific section on advocacy to NTD masterplans can facilitate aligning advocacy activities to the specific requirements and priorities required to achieve the objectives stated in the master plans as well as identifying opportunities for cross-cutting collaboration across programmes. The development of an integrated document can likewise help to avoid work repetition and incorporate advocacy activities into relevant reviews of programmatic progress and resource requirements (e.g., mid-term evaluations). |
| 1. **Communication strategies to enhance advocacy efforts and facilitate integration** |
| - **Support the use of the ‘multiple disease elimination’ concept to gather support from governments and international stakeholders for ‘last mile’ activities.** Programmes aiming for elimination often struggle to gather support since the disease burden for those NTDs has decreased and the cost of interventions in remaining hotspots can be high. There are opportunities for cross-NTD cooperation to advocate for additional support by presenting more ambitious and comprehensive objectives. - **Use evidence-based advocacy to support and raise awareness on elimination for NTDs.** Programmes should compile evidence about the multiple benefits of achieving elimination, health, economic or others, to showcase why last mile activities are crucial and worth investing in. - **Ensure advocacy campaigns across the sector are accompanied by work plans integrating clear objectives as well as cost-benefit estimates.** To be effective in mobilising additional support, advocacy campaigns should have concrete work plans that can be presented to funders, once they agree to discuss their potential engagement. Funders require a clear description of what the funds are required for, concrete information on the benefits that can be attained through proposed interventions (health, economic and others), as well as a clear account of the economic value of the activities proposed. - **Encourage programmes to identify and showcase the contributions of NTD interventions to public health and non-health sectors (e.g., WASH) in their advocacy efforts for inter-sectoral collaboration.** Campaigns should have a clear narrative that demonstrates the value of collaboration (e.g., health or economic benefits) to approach other sectors. Such evidence can help inter-sectoral advocacy work requesting support to scale-up operations. - **Foster the adoption of synergistic messages and frameworks during WHO-led high-level discussion forums.** Advocacy initiatives pressing for programme or disease-centered messages or agendas can confound stakeholders’ understanding of priorities and limit the possibility for cross-cutting partnerships, while affecting resource mobilisation for integrated initiatives. Greater coordination between countries and programmes to promote shared messages or adopt more holistic frameworks (e.g., One Health), according to their common concerns, may enable more collaborative discussions during high-level partners’ meetings. - **Encourage NTD programmes to make a case for cross-cutting initiatives centered on specific shared priorities,** as compared to using broad narrative under the ‘NTD’ label. It has been observed that ‘NTDs’, as a theme aggregating multiple diseases, regions, and intervention strategies, can face difficulties in gathering track among funders and policy makers. A more granular approach to integration around specific countries, subjects, or diseases may help to develop more compelling and clearer advocacy messages. |
| 1. **Strategic themes for advocacy initiatives on inter-sectoral collaboration** |
| - **Support advocacy initiatives showcasing the contribution of NTD programmes to child and maternal health**, **to support the development of formal mechanisms of collaboration and integration with public health systems.** These messages can be reinforced by highlighting the risk of not acting on this subject, both in terms additional health complications among women and children and oversights in technical debates and innovation (e.g., new drug formulations). This form of integration can foster the development of an integrated package of intervention to tackle multiple diseases (communicable and non-communicable) among vulnerable groups. Such collaboration may also allow NTD programmes to access additional funding and resources. - **Aim to position NTDs’ research and development agenda within emerging initiatives developed in response the COVID-19 pandemic.** There have been investments to enhance pandemic preparedness across several regions, which has resulted in greater local R&D capacity. Programmes can advocate for NTDs to have access to those facilities or equipment, which may otherwise not be given continuous use. - **Encourage advocacy efforts showcasing the role of NTD programmes in research and development, to promote collaboration with science and technology stakeholders.** NTD programmes provide a valuable platform for testing new technologies, conducting innovative experiments, and building local capacity in R&D (material and human). Advocacy initiatives can seek support for intersectoral collaboration, including with the commercial sector, by showcasing compelling evidence or case studies on how new technologies improve interventions’ health outcomes, equity, or cost-efficiency. These efforts may include discussing the use of digital tools, like Artificial Intelligence, for inter-sectoral collaboration.   NTD programmes can likewise operate as key platforms for developing and validating new treatments. R&D advocacy should identify opportunities for synergies, shared services, and integration with pharmaceutical companies and drug manufacturers. This may facilitate better, more equitable, and sustained access to treatments.   - **Outline an agenda and advocate for closer collaboration between NTDs and climate change initiatives.** Climate is a key driver of epidemiological trends for multiple NTDs. As climate change occurs, setbacks are likely. Certain NTDs may return after elimination has been achieved, while others may enter new territories, potentially becoming global outbreaks. Programmes should assess their vulnerability to climate change and seek support for collaborative R&D and plan coordinated activities with climate change initiatives. This is essential so that programmes can adopt measures to ensure equitable access to resources to address the needs of the most vulnerable, which are likely to be the most affected. - **Assess NTD programmes’ vulnerability to urban expansion and advocate for closer collaboration with urban planners and public health stakeholders.** Growing urbanisation in many countries generates the risk of NTD outbreaks across the globe. NTD programmes should pursue greater advocacy efforts to be included in research and evaluation projects assessing the potential health impacts of urbanisation as well as in policy debates discussing the public health response required to prevent or mitigate such impacts. |
